# Nitrogen recycling by the gut microbiome in sarcopenia

**DOI:** 10.3389/fmicb.2025.1698437

**Published:** 2026-01-05

**Authors:** Rosa Haller, Olha Hazia, Nicole Feldbacher, Julia Traub, Tobias Madl, Hansjörg Habisch, Angela Horvath, Vanessa Stadlbauer

**Affiliations:** 1 Division of Gastroenterology and Hepatology, Department of Internal Medicine, Medical University of Graz, Graz, Austria; 2 Center for Biomarker Research in Medicine (CBmed), Graz, Austria; 3 Department of Clinical Medical Nutrition, University Hospital Graz, Graz, Austria; 4 Otto Loewi Research Center, Medicinal Chemistry, Medical University of Graz, Graz, Austria; 5 BioTechMed-Graz, Graz, Austria

**Keywords:** cirrhosis, sarcopenia, gut microbiome, urease, nitrogen recycling, host–microbiome interaction

## Abstract

**Introduction:**

Sarcopenia, which is defined as loss of skeletal muscle mass and strength, affects up to 70% of patients with liver cirrhosis. Since hibernating animals maintain muscle mass through microbial nitrogen recycling, urease-producing bacteria may have a protective role in humans. We hypothesized that altered microbial urease abundance contributes to differences in nitrogen recycling potential between patients with and without sarcopenia, with sex-specific effects.

**Methods:**

Stool samples from 152 patients with (*n* = 101) and without sarcopenia (*n* = 51) were analyzed. Functional profiles were predicted from 16S rRNA gene amplicon sequencing data using Tax4Fun2, and predicted abundances of urease subunit alpha were extracted. A systematic literature search identified 120 urease-producing taxa, of which 35 were represented in sequencing data.

**Results:**

Sarcopenia is associated with a lower predicted abundance of urease subunit alpha in patients with cirrhosis (*n* = 96; *p* = 0.045, *r* = 0.20; median = 0.0002 vs. 0.0004), irrespective of sex, and in women (*n* = 49, *p* = 0.037, *r* = 0.30, median = 0.0002 vs. 0.0004), irrespective of cirrhosis. Urease subunit alpha abundance increases with the use of proton pump inhibitors (PPIs) in the entire patient cohort (*p* = 0.0028, *r* = 0.24, median = 0.0003 vs. 0.0002), patients with cirrhosis (*p* = 0.033, *r* = 0.22, median = 0.0004 vs. 0.0002), and men (*n* = 103, *p* = 0.0005, *r* = 0.34, median = 0.0002 vs. 0.0001). Beta-blockers are associated with higher urease subunit alpha abundance in the entire patient cohort (*p* = 0.018, *r* = 0.19, median = 0.0003 vs. 0.0002) and women (*p* = 0.031, *r* = 0.31, median = 0.0004 vs. 0.0002). The overall abundance of potentially urease-producing taxa was comparable between the groups.

**Discussion:**

The increased urease subunit alpha abundance in patients with liver cirrhosis and women without sarcopenia, and the influence of medication on abundance, point towards potential additional effects of beta-blockers in sarcopenia.

## 1 Introduction

Sarcopenia was first described as a decline in lean body mass, which affects mobility (Rosenberg, 1997). Muscle function was a better predictor of mortality in patients with sarcopenia than muscle mass, and was introduced into consensus definitions of sarcopenia (Newman et al., 2006; Morley et al., 2011). In liver cirrhosis, the 10th most common cause of death in the Western world, up to 70% of patients are affected by sarcopenia (Kim et al., 2017; Ginès et al., 2021). Sarcopenia increases the mortality risk of patients with cirrhosis (Tantai et al., 2022). Like cirrhosis, sarcopenia is more prevalent in men (Lai et al., 2021). No medical treatment for sarcopenia is available, and strategies like exercise and nutritional supplements are of limited success (Ebadi et al., 2023). The main reason for the lack of specific treatment is the incomplete understanding of the pathophysiology of sarcopenia in cirrhosis.

Hibernating mammals are often studied in muscle research since several species do not experience extensive muscle loss during hibernation. Understanding the mechanisms that protect hibernating mammals from muscle loss could also help develop improved treatment strategies for patients with sarcopenia. Nitrogen recycling has long been proposed as one of the mechanisms protecting hibernating mammals from muscle loss. In this process, urea produced by the body is hydrolyzed by the gut microbiome to ammonia. The resulting nitrogen is used to synthesize amino acids, which are incorporated into proteins formed subsequently (Nelson et al., 1975; Rice et al., 2020). These observations highlight the need for translational studies to understand whether similar mechanisms occur in humans.

Recently, Regan et al. brought more clarity to the process of nitrogen recycling during hibernation in thirteen-lined ground squirrels (*Ictidomys tridecemlineatus*). They could show that fasting deprives squirrels of dietary nitrogen during hibernation, risking protein imbalance. Despite nitrogen deficiency and inactivity, the squirrels lose little muscle mass or function—a finding the researchers linked to a higher percentage of urease genes in the gut metagenome of hibernating squirrels. Additionally, they proved that the produced nitrogen was then incorporated into the squirrels’ protein (Regan et al., 2022).

Urease is an amidohydrolase (EC 3.5.1.5), which can be found in various bacterial species (Suzuki et al., 1979). The enzyme catalyzes the hydrolysis of urea to ammonia and carbamate. The latter decomposes to an additional ammonia molecule and carbonic acid. This leads to an increase in pH in the environment of the urease-producing microorganism, which is important for bacterial survival (Mora and Arioli, 2014). While urease is classified as a virulence factor, it is also expressed by bacteria with anti-inflammatory properties (Mora and Arioli, 2014; Savary-Auzeloux et al., 2022). Urease expression by the gut microbiome is important for human health, as up to 30% of the produced urea is hydrolyzed by microorganisms (Walser and Bodenlos, 1959).

Gut microbiome alterations of patients with cirrhosis are well described, showing an impact on the prognosis of liver disease and infection risk after transplantation (Qin et al., 2014; Vieira and Baumert, 2024). The composition and function of the gut microbiome are altered in patients suffering from sarcopenia, depending on the underlying disease (Wang et al., 2022; Aliwa et al., 2023). Additionally, various medications [e.g., proton pump inhibitors (PPIs)] are known to influence the gut microbiome (Horvath et al., 2019; Forslund et al., 2021).

However, the role of nitrogen recycling in the gut microbiome and its effects on sarcopenia and liver cirrhosis is yet unknown. We hypothesize that gut microbial nitrogen recycling could benefit humans and help maintain muscle mass and function. Therefore, we aim to gain a better understanding of nitrogen recycling by the gut microbiome in sarcopenia and cirrhosis, with a special focus on interaction with drugs and sex-specific differences.

## 2 Methods

### 2.1 Study protocol

Patients with and without liver cirrhosis were recruited between April 2017 and January 2019 at the Medical University of Graz. The study is registered at clinicaltrials.gov (NCT03080129) and was approved by the research ethics committee of the Medical University of Graz (29–280 ex 16/17). It was conducted with informed consent, in accordance with the principles of the Declaration of Helsinki. In the cirrhotic group, men and women over 18 years, who gave written consent and had a diagnosis of liver cirrhosis (clinical/radiological and computed tomography/magnetic resonance imaging [CT/MRI]). We excluded patients with hepatic encephalopathy > grade 2 and other cognitive disorders, hepatocellular carcinoma stage Barcelona Clinic Liver cancer stage C or D, ursodeoxycholic acid (UDCA) treatment, intake of pro- and antibiotics, and lack of written consent. Men and women aged 18 or older who provided written consent and underwent a CT/MRI scan were included in the control group. Exclusion criteria were a diagnosis of cirrhosis, disorders not allowing informed consent, and current intake of pro- and anti-infective antibiotics. Sarcopenia was diagnosed per the European Working Group of Sarcopenia in Older People (EWGSOP) 2010 criteria, which was shown to be the most suitable for patients with liver cirrhosis (Cruz-Jentoft et al., 2010; Aliwa et al., 2023). The exact cut-offs for this study are shown in Supplementary Table S1. The risk of hepatic encephalopathy (HE) was calculated using the score developed by Tapper et al. (2018). The entire patient cohort (*n* = 178) was previously described in more detail (Aliwa et al., 2023).

### 2.2 Microbiome analysis

The gut microbiomes of 156 patients were analyzed using 16S ribosomal RNA (rRNA) gene amplicon sequencing. DNA was isolated using a MagNA Pure LC DNA isolation kit (Roche, Mannheim, Germany) according to the manufacturer’s instructions. V1-2 were amplified (forward primer: AGAGTTTGATCCTGGCTCAG, reverse primer: TGCTGCCTCCCGTAGGAGT) and sequenced using Illumina MiSeq technology (Illumina, Eindhoven, Germany). Sequencing quality was confirmed with positive and negative controls (Stadlbauer et al., 2017; Aliwa et al., 2023). Sequence reads were pre-processed on QIIME2 on a local Galaxy server (https://galaxy.medunigraz.at). Denoising was performed with DADA2. Taxonomy was assigned based on the Silva V132 database with a Naïve Bayes classifier. Sequencing data is available in the NCBI Sequencing Read Archive (PRJNA933898, https://www.ncbi.nlm.nih.gov/sra/PRJNA933898) (Aliwa et al., 2023). BioSample IDs are listed in Supplementary Table S2. The amplicon sequence variant (ASV) table obtained, including taxonomic information, was imported into R for further analysis. Cyanobacteria and patients with <8,000 sequence reads were excluded from the analysis (*n* = 4). We predicted the functional profiles of microbial communities based on 16S rRNA gene amplicon sequencing data (Tax4Fun2) (Wemheuer et al., 2020). We extracted the predicted abundance of urease subunit alpha as a representation of the abundance of urease. Patients with <8,000 sequence reads were excluded from the analysis.

### 2.3 NMR analysis

Serum and stool samples were prepared as described previously (Kreuzer et al., 2022). Briefly, they were mixed with 66% methanol in water. Following centrifugation, the supernatant was lyophilized and resuspended with a buffer solution [0.08 M Na_2_HPO_4_ in D_2_O, pH 7.4, 4.6 mM trimethylsilyl propionic acid (TMSP)] and quantified at 310 K using a NMR spectrometer (Bruker Avance 600 MHz, limit of detection = 1 μM/L). The acquired spectra of 105 patients were normalized using probabilistic quotient normalization and metabolite integrals, and the resulting data were analyzed in MetaboAnalyst 6.0 (www.metaboanalyst.ca, last accessed 02.02.2024). In total, 47 metabolites in stool and 42 metabolites in serum were measured (Supplementary Table S3). For a more in-depth analysis, we analyzed metabolites related to muscle health: short-chain fatty acids (Han et al., 2022), proteinogenic amino acids, non-proteinogenic amino acids, metabolites involved in amino acid metabolism, metabolites involved in muscle health, and vitamins.

### 2.4 Systematic literature search

To further evaluate the role of urease in the gut microbiome, a systematic literature search was performed in PubMed on 20 February 2023 using the keywords “UREASE” and “GUT,” excluding “HELICOBACTER.” All records were screened for eligibility, and only studies reported in English were included. The PubMed search resulted in 110 records.

The records were manually reviewed for urease-producing taxa. The abundance of the identified taxa was tested in our dataset of patients with and without sarcopenia. Taxa above family level, genera that were not closely enough defined in the literature, were excluded from the analysis. Taxa occurring at a very low frequency (<10% of each analyzed group) were omitted from further analysis.

### 2.5 Statistical analysis

The dataset was split into patients with and without cirrhosis to determine a possible cirrhotic-specific effect. As both diseases are more common in men, we analyzed the effect of sex and sarcopenia on urease abundance and the sexes separately to study sex-specific differences. Comparative statistics (Mann–Whitney U test) were used to compare the abundance of urease subunit alpha between patients with and without cirrhosis, and men and women with and without sarcopenia. In our analysis, we included the medications used by more than 20% of patients. Medication with a significant effect were also tested for their interaction in a linear model. Features showing a significant difference between groups were analyzed using linear models adjusted for age and body mass index (BMI). In patients with liver cirrhosis, the Graz malnutrition screening (GMS) (Roller et al., 2016). The Model for End-Stage Liver Disease (MELD) score was additionally included in the analysis.

Comparative statistics (Mann–Whitney U test) were used to compare the abundance of urease-producing taxa identified in our literature review between patients with and without cirrhosis, and men and women with and without sarcopenia. The correlation was assessed using Spearman’s rank–order test.

The *p*-values obtained from comparative tests between bacterial taxa and from correlation analyses were corrected with the Benjamini–Hochberg method. Statistical analysis was performed in R (version 4.3.3). All packages used are cited in the supplements. All figures were created in R Studio.

## 3 Results

### 3.1 Patient characteristics

A total of 152 patients were included in this analysis after exclusion of samples with low sequence reads (*n* = 4), 96 with liver cirrhosis (64 with sarcopenia, 32 without), and 56 without liver cirrhosis (37 with sarcopenia, 19 without). In total, 103 of the patients were men (73 with sarcopenia, 30 without) and 49 were women (28 with sarcopenia, 21 without). The demographic data are presented in Table 1, and the markers of muscle function are described in Table 2.

**Table 1:** Patient characteristics of the analyzed groups.

	Patients									
	All patients (*n* = 152)		Cirrhotics (*n* = 96)		Non-cirrhotics (*n* = 56)		Men (*n* = 103)		Women (*n* = 49)	
Sarcopenia (yes/no)	101 (67%)	51 (33%)	64 (67%)	32 (33%)	37 (66%)	19 (34%)	73 (71%)	30 (29%)	28 (57%)	21 (43%)
Cirrhosis (yes/no)	64 (63%)/37 (37%)	32 (63%)/19 (37%)	–		–		54 (74%)/19 (26%)	17 (57%)/13 (43%)	10 (36%)/18 (64%)	15 (71%)/6 (29%)
Age	64 (61; 66)	62 (57, 64)	65.5 (61.0; 68.0)	62 (56; 67)	59 (56; 66)	58 (52; 67)	64 (61; 68)	62 (57; 68)	61 (54; 66)	58 (51; 66)
Sex (m/f)	73 (72%)/28 (29%)	30 (59%)/21 (41)	54 (84%)/10 (16%)	17 (53%)/15 (47%)	19 (51%)/18 (49%)	13 (68%)/6 (32%)	–		–	
PPIs (yes/no)	44 (44%)/57 (51%)	23 (45%)/28 (55%)	33 (52%)/31 (48%)	17 (53%)/15 (47%)	11 (30%)/26 (70%)	13 (68%)/6 (32%)	34 (47%)/39 (53%)	12 (40%)/18 (60%)	10 (36%)/18 (64%)	11 (52%)/10 (48%)
Beta-blocker (yes/no)	49 (49%)/52 (51%)	23 (45%)/28 (55%)	41 (64%)/23 (36%)	18 (56%)/13 (44%)	8 (22%)/29 (78%)	4 (21%)/15 (79%)	41 (56%)/32 (44%)	12 (40%)/18 (60%)	8 (29%)/20 (71%)	11 (52%)/10 (48%)
Statins (yes/no)	10 (10%)/91 (90%)	10 (20%)/41 (80%)	5 (8%)/59 (92%)	6 (19%)/26 (81%)	5 (14%)/32 (86%)	4 (21%)/15 (79%)	6 (8%)/67 (92%)	7 (23%)/23 (77%)	4 (14%)/24 (86%)	3 (14%)/18 (86%)
Diuretics (yes/no)	42 (71%)/59 (29%)	24 (47%)/37 (53%)	39 (61%)/25 (39%)	14 (44%)/18 (56%)	3 (8%)/34 (92%)	0 (0%)/19 (100%)	35 (48%)/38 (52%)	7 (23%)/23 (77%)	7 (25%)/21 (75%)	7 (33%)/14 (67%)
BMI	25 (24; 26)	29 (28; 30)	26 (24; 28)	30 (27; 33)	24 (22; 25)	28 (25; 30)	26 (25; 27)	29 (28; 31)	22 (20; 25)	30 (24; 33)
Child grade (A/B/C)	–		27 (42%)/29 (45%)/8 (13%)	18 (56%)/9 (28%)/5 (16%)	–		25 (46%)/23 (43%)/6 (11%)	11 (65%)/5 (29%)/1 (6%)	2 (22%)/6 (56%)/2 (22%)	7 (46%)/4 (27%)/4 (27%)
MELD	–		11 (9; 13)	10 (8; 12)	–		10 (9; 13)	10 (8; 11)	11 (8; 27)	12 (8; 20)

**Table 2:** Parameters of muscle function and muscle biomarkers for the analyzed groups.

	Patients									
	All patients (*n* = 152)		Cirrhotics (*n* = 96)		Non-cirrhotics (*n* = 56)		Men (*n* = 103)		Women (*n* = 49)	
Sarcopenia (yes/no)	101 (67%)	51 (33%)	64 (67%)	32 (33%)	37 (66%)	19 (34%)	73 (71%)	30 (29%)	28 (57%)	21 (43%)
Muscle function										
Muscle mass (cm^2^/m^2^)										
Handgrip strength (kg)	29.30 (26.70; 31.60)	31.00 (27.30; 35.70)	29.32 (27.00; 31.70)	28.18 (24.30; 33.60)	27.3 (22.0; 36.0)	40.0 (29.0; 48.6)	31.7 (29.5; 35.0)	35.85 (31.50; 41.00)	21.1 (18.60; 23.60)	24.0 (21.00; 27.66)
Mid-arm muscle circumference (cm)	237.30 (231.30; 247.20)	270.47 (259.13; 280.88)	236.72 (230.55; 251.98)	273.94 (247.73; 282.95)	240.49 (208.42; 254.86)	265.44 (250.00; 284.29)	245.44 (235.90; 255.49)	275.75 (264.24; 282.95)	208.29 (202.87;241.72)	251.0 (231.03; 280.88)
Triceps skinfold thickness (mm)	12.25 (11.0; 14.20)	14.20 (11.00; 15.00)	12.05 (11.00; 14.60)	14.05 (10.00; 15.10)	12.80 (9.50; 16.20)	14.60 (9.00; 17.60)	11.65 (10.10; 13.20)	14.20 (10.0; 17.0)	15.50 (10.60; 18.00)	13.90 (10.00; 15.00)
Gait speed (m/s)	0.96 (0.88; 1.0)	1.03 (0.98; 1.13)	0.94 (0.84; 1.03)	1.03 (0.87; 1.16)	1.00 (0.86; 1.14)	1.05 (0.98; 1.20)	0.97 (0.91; 1.07)	1.04 (0.98; 1.19)	0.88 (0.80; 1.09)	1.03 (0.87; 1.09)
Chair raise (s)	17.6 (16.5; 19.2)	15.5 (14.02; 17.50)	18.49 (16.84; 20.32)	15.84 (12.66; 20.06)	16.69 (14.16; 19.37)	15.13 (12.69; 17.09)	17.69 (16.41; 19.60)	14.41 (12.69; 17.53)	17.44 (13.16; 19.66)	16.65 (12.09; 19.28)
Muscle biomarker										
Myostatin (ng/ml)	36.96 (35.48; 39.50)	43.07 (36.21; 46.77)	36.12 (33.80; 42.02)	41.62 (29.24; 46.64)	38.59 (35.39; 40.90)	45.63 (36.21; 60.26)	38.63 (35.51; 42.82)	45.63 (36.21; 57.16)	36.19 (32.81; 38.55)	36.84 (28.48; 46.23)
Irisin (μg/ml)	1.89 (1.62; 2.19)	1.98 (1.67; 2.27)	1.66 (1.35; 2.05)	1.82 (1.33; 2.19)	2.58 (1.9; 3.59)	2.27 (1.85; 3.55)	1.83 (1.47; 2.12)	2.11 (1.81; 2.75)	2.15 (1.56; 3.49)	1.82 (1.22; 2.60)
FGF-21 (ng/ml)	0.35 (0.25; 0.41)	0.25 (0.15; 0.36)	0.36 (0.23; 0.48)	0.24 (0.09; 0.37)	0.33 (0.24; 0.42)	0.25 (0.12; 0.43)	0.31 (0.22; 0.41)	0.27 (0.13; 0.39)	0.37 (0.25; 0.50)	0.24 (0.08; 0.37)
IGF-1 (ng/ml)	78.66 (63.85; 105.41)	87.67 (8.15; 110.33)	58.30 (43.55; 67.36)	51.09 (31.95; 82.56)	134.54 (117.10; 159.61)	114.43 (101.31; 178.99)	65.70 (54.70; 90.80)	89.96 (47.79; 119.23)	113.66 (74.62; 157.31)	76.35 (50.43; 114.43)

### 3.2 Prediction of urease gene abundance in functional profiles of microbial communities based on 16S rRNA gene amplicon sequencing

**Figure 1:**
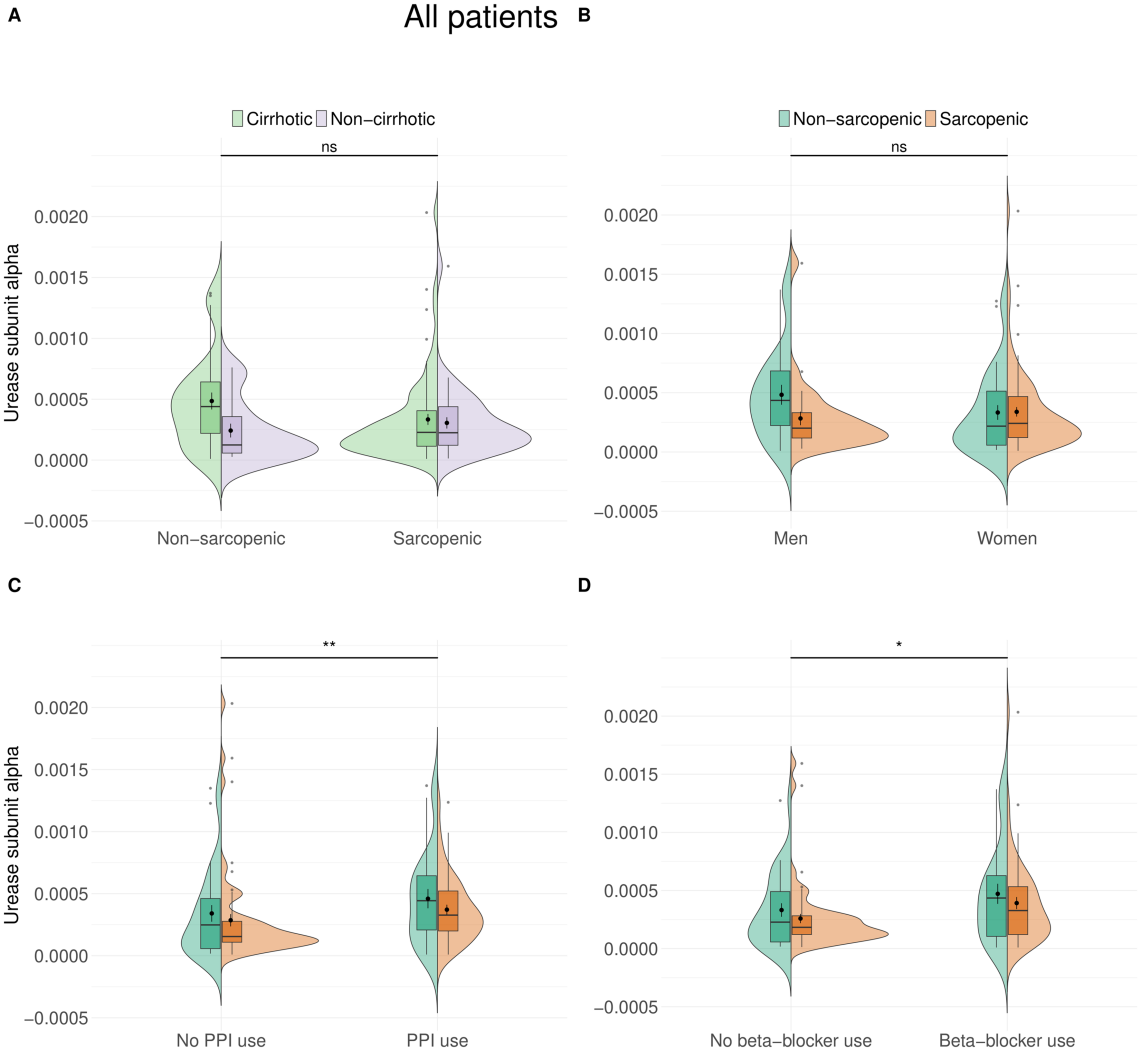
There is no difference in urease abundance between patients with and without sarcopenia in the entire cohort **(A)**, nor is there a difference depending on sex **(B)**. Urease abundance was increased in both patients who took PPIs (*n* = 67, *p* = 0.0028) **(C)** and beta-blockers (*n* = 71, *p* = 0.018) **(D)**.

There was no difference in urease subunit alpha abundance between patients with and without sarcopenia, and neither was there a difference between patients with and without cirrhosis or between sexes (Figures 1A,B). Interestingly, we found that urease abundance was higher in all patients who took proton pump inhibitors (PPIs) (*p* = 0.0028, *r* = 0.24, median = 0.0003 vs. 0.0002) and in patients who took beta-blockers (*p* = 0.018, *r* = 0.19, median = 0.0003 vs. 0.0002) (Figures 1C,D). The influence of PPI intake did not remain significant after covariates were included in the analysis. Beta-blocker, on the other hand (*β* = 1.20 × 10^−4^, *p* = 0.035), remained significantly associated with urease subunit alpha after adjustment for age and BMI. However, the association between age and BMI, and the overall model, was not significant. Testing PPIs and beta-blockers as covariates, only beta-blockers show a significant positive association with abundance (*β* = 1.172 × 10^−4^, *p* = 0.038).

**Figure 2:**
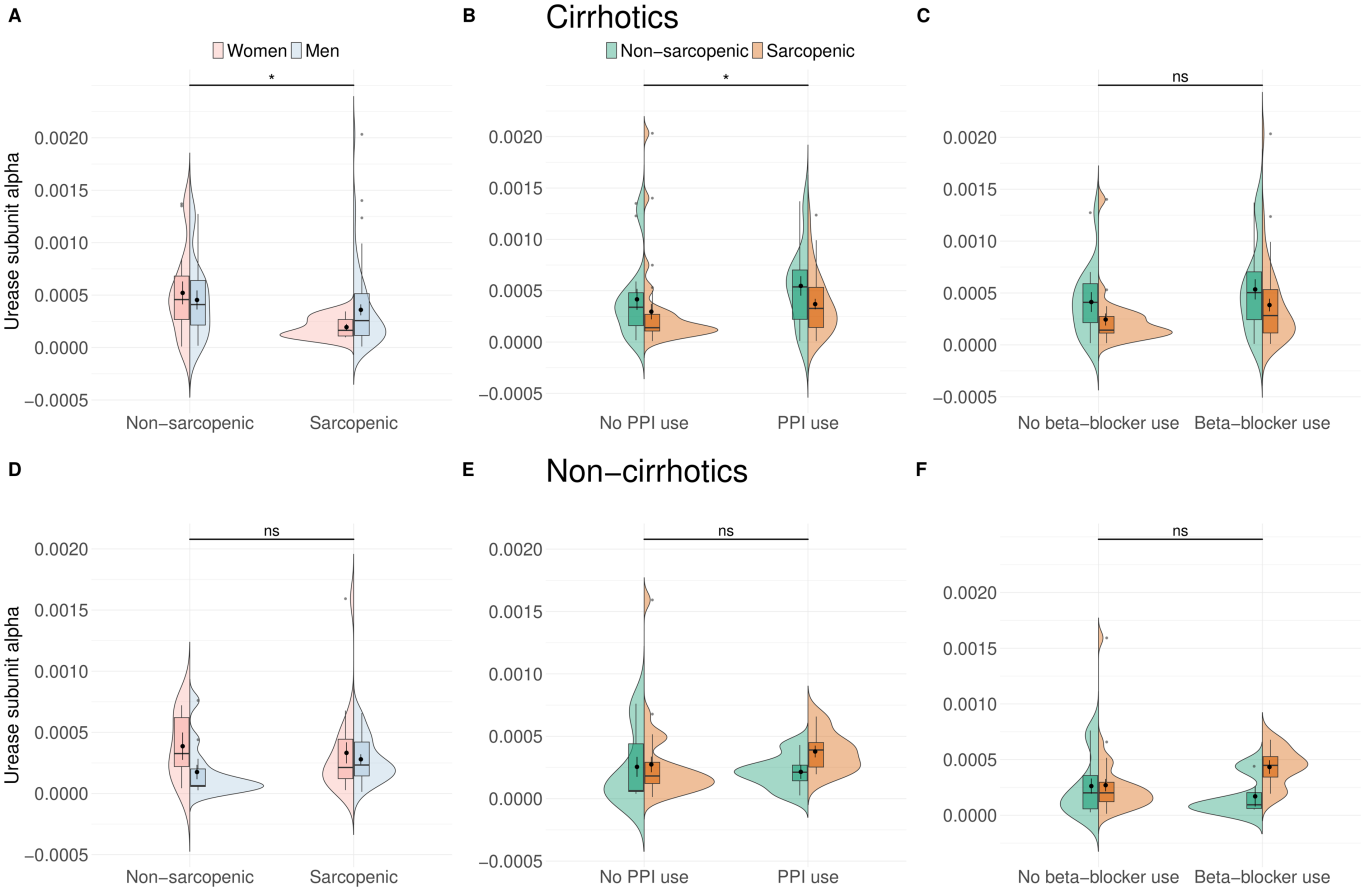
Urease abundance was decreased in patients with cirrhosis and with sarcopenia, independent of sex (*n* = 64, *p* = 0.033) **(A)**. PPIs use increased urease abundance in patients with cirrhosis (*n* = 67, *p* = 0.033) **(B)**, whereas beta-blockers did not have an influence **(C)**. Urease abundance was not changed in patients without cirrhosis **(D–F)**.

When examining cirrhosis, we found lower urease abundance in patients with sarcopenia than in those without (*p* = 0.045, *r* = 0.20; median = 0.0002 vs. 0.0004) (Figure 2A). After covariate analysis, none of the predictors were significantly associated with urease subunit alpha abundance. As in the whole cohort, urease subunit alpha abundance was higher in patients with cirrhosis who took PPIs (*p* = 0.033, *r* = 0.22, median = 0.0004 vs. 0.0002) (Figure 2B). After analysis, including age, BMI, MELD score, and GMS, neither variable was found to be significantly associated with urease subunit alpha. There was no difference in urease subunit alpha abundance among patients with liver cirrhosis, regardless of beta-blocker intake (Figure 2C). Neither PPIs nor beta-blockers showed a significant association with urease subunit alpha abundance in a linear model.

There was no correlation between urease abundance and the HE risk score (*r*_s_ = −0.086, *p* = 0.41). In patients without cirrhosis, we found no association between urease subunit alpha abundance and sarcopenia status, by sex or medication (Figures 2D,F).

Interaction testing between sex and sarcopenia state showed lower urease abundance in women with sarcopenia (*β* = 1.20 × 10^−4^, *p* = 0.044); however, there was no significant interaction between sex and sarcopenia.

**Figure 3:**
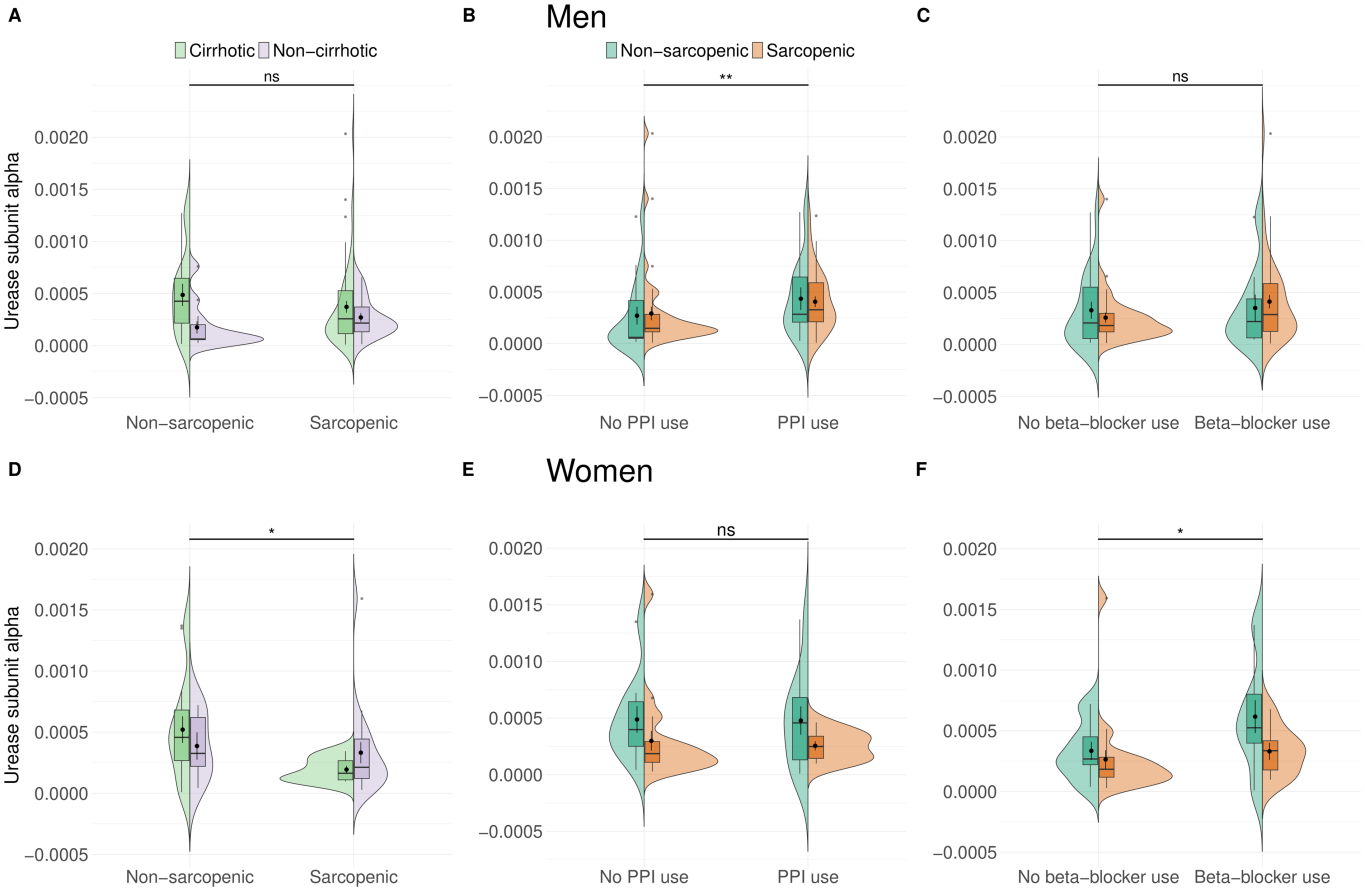
In men, there was no difference in urease abundance between patients with and without sarcopenia **(A)**. Urease was increased in men who took PPIs (*n* = 46, *p* = 0.0005) **(B)**, but not beta-blocker **(C)**. In women, urease abundance was decreased in patients without sarcopenia (*n* = 28, *p* = 0.037) **(D)**. PPIs did not influence urease abundance **(E)**, but women who took beta-blockers had an increased abundance of urease (*n* = 19, *p* = 0.031) **(F)**.

When looking at sex differences in more detail, men showed no association between urease subunit alpha abundance and sarcopenia or the presence of cirrhosis (Figure 3A). However, urease subunit alpha abundance was significantly higher in men who took PPIs (*p* = 0.0005, *r* = 0.34, median = 0.0002 vs. 0.0001) (Figure 3B). When adding the covariates age and BMI, PPIs were significantly positively associated with urease subunit alpha (*β* = 1.36 × 10^−4^, *p* = 0.043), while age, BMI, and the overall model were not significant. Beta-blocker intake did not affect urease subunit alpha abundance in men (Figure 3C). PPIs and beta-blockers did not show an association with urease subunit alpha abundance in a linear model.

In women, urease subunit alpha abundance was decreased in those with sarcopenia (*p* = 0.037, *r* = 0.30, median = 0.0002 vs. 0.0004), irrespective of cirrhosis or PPI intake (Figures 3D,E). After adjusting for age and BMI, sarcopenia was significantly associated with lower urease subunit alpha abundance (*β* = −2.50 × 10^−4^, *p* = 0.039), while age, BMI, and the model were not significant. Adding PPIs and beta-blockers as covariates, only beta-blockers show a significant positive association with abundance (*β* = 2.312 × 10^−4^, *p* = 0.034).

Urease subunit alpha abundance was higher in women who took beta-blockers (*p* = 0.031, *r* = 0.31, median = 0.0004 vs. 0.0002) (Figure 3F). With age and BMI as covariates, beta-blocker intake was significantly higher than urease subunit alpha abundance (*β* = 2.17 × 10^−4^, *p* = 0.049). Age, BMI, and the model, however, were not significant.

In neither group did the abundance of urease subunit alpha differ between patients taking diuretics (Supplementary Figure S1), nor did it correlate with markers for muscle function, muscle biomarkers, or protein production markers in serum or serum urea (Supplementary Figure S2; Supplementary Table S4).

To further investigate the influence of urease subunit alpha on protein production, we correlated predicted urease subunit alpha abundance with a set of metabolites in stool and serum. We analyzed metabolites that are potential markers for muscle health, protein production, nutrition, and a healthy microbiome. The analyzed metabolites included amino acids, metabolites involved in amino acid metabolism, muscle metabolism, vitamins, and short-chain fatty acids. No correlation was found between the chosen metabolites and urease subunit alpha (Supplementary Figure S3; Supplementary Table S5).

### 3.3 Systematic literature analysis

Of the 110 identified records, 43 were included in the analysis. In total, 126 taxa were identified in the literature search; 40 taxa were present in the dataset and could be extracted, and 23 taxa were present in more than 10% of the patients (Supplementary Figure S5; Supplementary Table S6).

**Figure 4:**
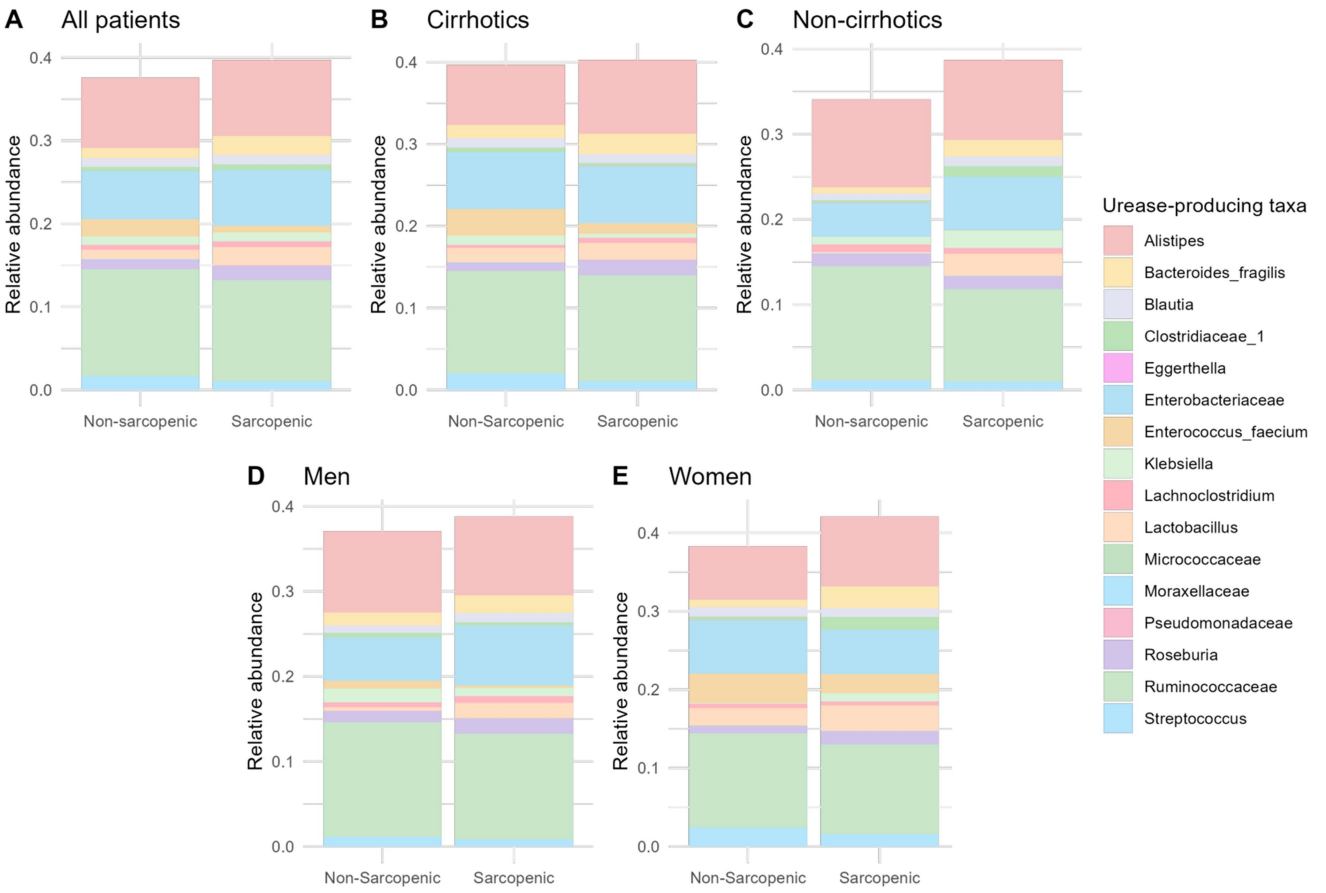
No difference in urease-producing taxa was found in the entire patient cohort **(A)**, patients with and without cirrhosis **(B,C)**; men **(D)**; or women **(E)**.

No difference in the abundance of the extracted taxa between the groups (sarcopenia/cirrhosis/sex) was observed (Figure 4; Supplementary Table S7).

The relative abundance of the extracted taxa was not associated with urease subunit alpha abundance (Supplementary Figure S4; Supplementary Table S8).

## 4 Discussion

In this study, we aimed to assess differences in bacterial urease gene abundance between patients with and without sarcopenia and the influence of two commonly used drugs (PPIs and beta-blockers) on patients with liver cirrhosis. Contrary to our expectations, the predicted urease abundance does not differ between patients with and without sarcopenia in the entire cohort.

While there was neither a difference in predicted urease subunit alpha abundance nor in the abundance of urease-producing taxa in relation to the underlying disease or sex in the entire patient cohort, urease subunit alpha abundance was increased in patients who took PPIs or beta-blockers. PPI intake is associated with increased oral bacterial taxa in the gut microbiome, such as *Streptococcus*, which is known to produce urease (Imhann et al., 2016). However, the intake of PPIs has been associated with muscle loss due to nutrient deficiency, such as vitamin B12, magnesium, and potassium, and alterations in the gut microbiome, which lead to increased inflammation (Mohn et al., 2018; Vinke et al., 2020) and adverse outcomes (Horvath et al., 2019). As we expected increased muscle mass due to urease-producing taxa, this finding contrasts our primary hypothesis. *Streptococcus* could increase the abundance of the urease subunit alpha, but this may not confer any benefit for muscle health. The influence of beta-blockers on urease abundance is independent of age and BMI. Furthermore, a positive effect on sarcopenia in patients with cirrhosis has been observed, which could be explained by a decreased risk for bacterial infections in patients taking beta-blockers (Krell et al., 2013; Merli et al., 2015; Li et al., 2021) and decreased intestinal permeability (Reiberger et al., 2013). Furthermore, it was shown that nebivolol reduced muscle loss in a rat model of chronic heart failure (Dalla Libera et al., 2010). To clarify the influence of beta-blockers on muscle mass in patients with heart failure, a systematic review was conducted. Still, it was unable to clarify the role of beta-blockers (Saied et al., 2024). Changes in the gut microbiome and gut-microbiota-derived metabolites were observed (Lin et al., 2021) and it was suggested that beta-blockers work as antagonists of the microbial metabolite phenylacetylglutamine (Nemet et al., 2020).

When only patients with cirrhosis were considered, urease subunit alpha abundance was decreased in those with sarcopenia, suggesting a potential defect in nitrogen recycling in cirrhosis. This effect was influenced by the severity of liver cirrhosis (MELD score) and malnutrition screening (GMS). Urease in cirrhosis may be a double-edged sword, as ammonia produced by urease is neurotoxic, contributes to the pathogenesis of hepatic encephalopathy (HE), and is predictive of hospitalizations and mortality (Tranah et al., 2022). Higher ammonia levels are associated with increased muscle loss, and in portacaval anastomosis rats, ammonia-lowering therapy improved lean body mass (Kumar et al., 2017). We unfortunately do not have ammonia levels available from our cohort, and therefore aimed to relate urease abundance to the HE risk score (Tapper et al., 2018). However, we were unable to find an association. The question, therefore, remains open as to whether microbial urease-produced ammonia influences blood ammonia levels. In arctic ground squirrels (*Urocitellus parryii*), it was suggested that nitrogen recycling buffers the ammonia toxicity by removing the free nitrogen and incorporating it into amino acids (Rice et al., 2020), a similar mechanism in patients with cirrhosis needs to be further investigated. Ammonia measurements should therefore be included in future studies. Similar to the entire cohort, there was no difference in sex, but urease subunit alpha abundance was increased in patients with PPI intake, which is also associated with an increased risk of HE and therefore is contrary to our expectations (Tsai et al., 2017).

When analyzing sex-specific differences, we found that in women with sarcopenia, predicted urease subunit alpha abundance is decreased. Interestingly, this was independent of liver cirrhosis, PPI intake, age, and BMI. However, beta-blocker intake was associated with increased urease subunit alpha abundance in women, and this association was not affected by covariates age and BMI. Sex-specific effects have long been suggested in cirrhosis, without reaching a conclusion (Burza et al., 2017). For future works, not only the previously discussed ammonium levels should be included, but also hormonal influences and dietary habits.

The limitations of our pilot study include the lack of transcriptomic analysis and prediction based on urease subunit alpha abundance, the small sample sizes in the subgroups, and the unavailability of data on the patients’ dietary habits and ammonia levels.

## 5 Conclusion

Patients with cirrhosis and sarcopenia, and women with sarcopenia, showed lower abundance of the levels of the urease subunit alpha gene in the gut microbiome. PPI use and beta-blocker use were associated with higher urease gene abundance. Overall, our results indicate that urease expression in the gut microbiome may be related to sarcopenia, depending on the underlying disease and sex, and may be influenced by drugs. This suggests a possible role of the gut microbiome in nitrogen recycling in humans. Differences in sex, underlying diseases, and the influence of medication may help identify a potential precision medicine target. Further investigations, including ammonia levels and transcriptomics, will be needed to better understand the role of nitrogen recycling in sarcopenia.

## Data Availability

The data analyzed in this study is subject to the following licenses/restrictions: the sequencing data are available in the NCBI Sequencing Read Archive (PRJNA933898, https://www.ncbi.nlm.nih.gov/sra/PRJNA933898). Upon reasonable request, the clinical data are also available from the corresponding author, Vanessa Stadlbauer. Requests to access these datasets should be directed to vanessa.stadlbauer@medunigraz.at.
