# Supplementary material for: Nitrogen recycling by the gut microbiome in sarcopenia
Source: Front Microbiol. 2026 Jan 5;16:1698437. doi: 10.3389/fmicb.2025.1698437 (PMC12812614; doi:10.3389/fmicb.2025.1698437)
Supplement: Supplementary file 1 [file Data_Sheet_1.docx]

Supplementary Material

# Supplementary Figures


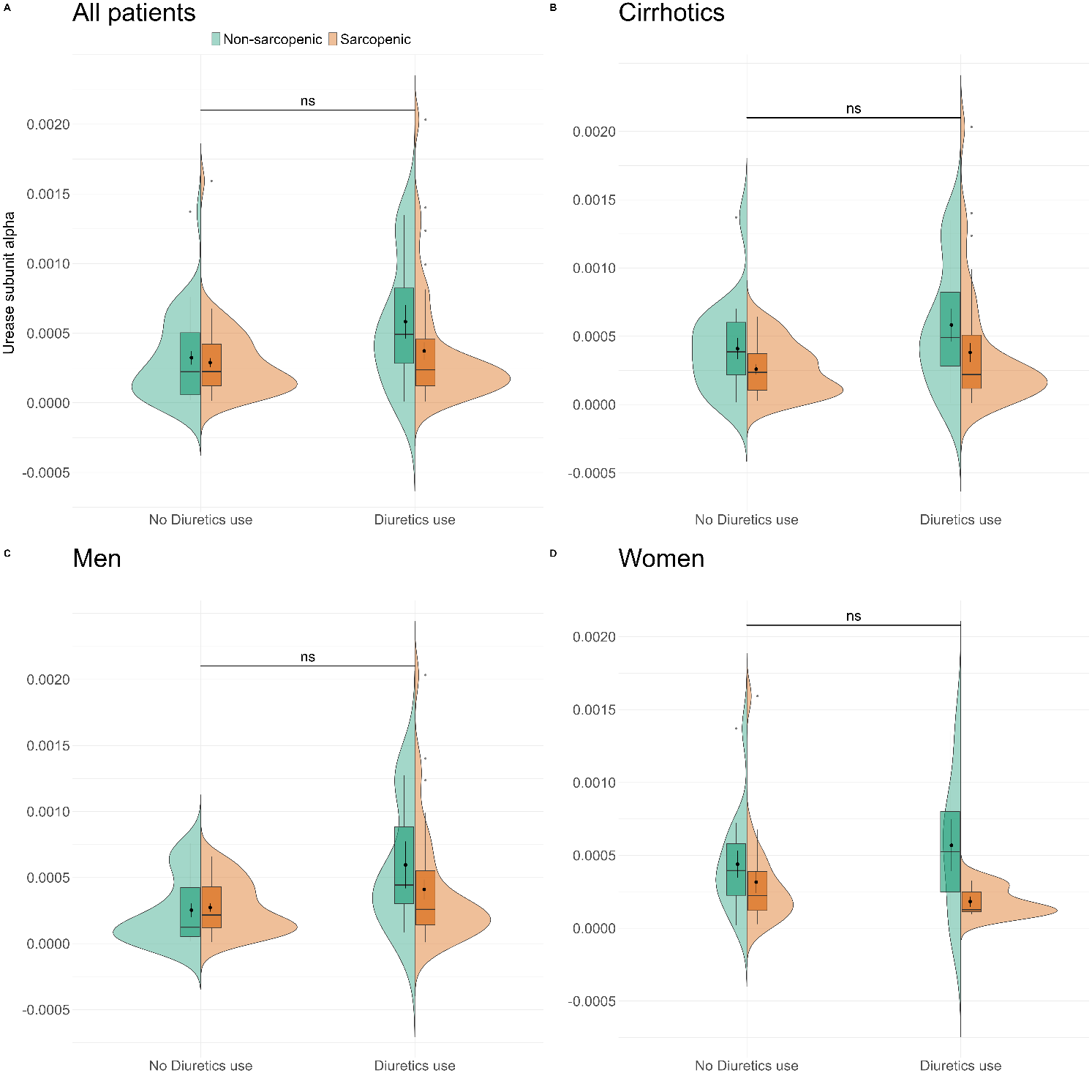
**Supplementary Figure S1:** There was no significant difference in urease abundance between patients who did not and did take diuretics for all analyzed groups.


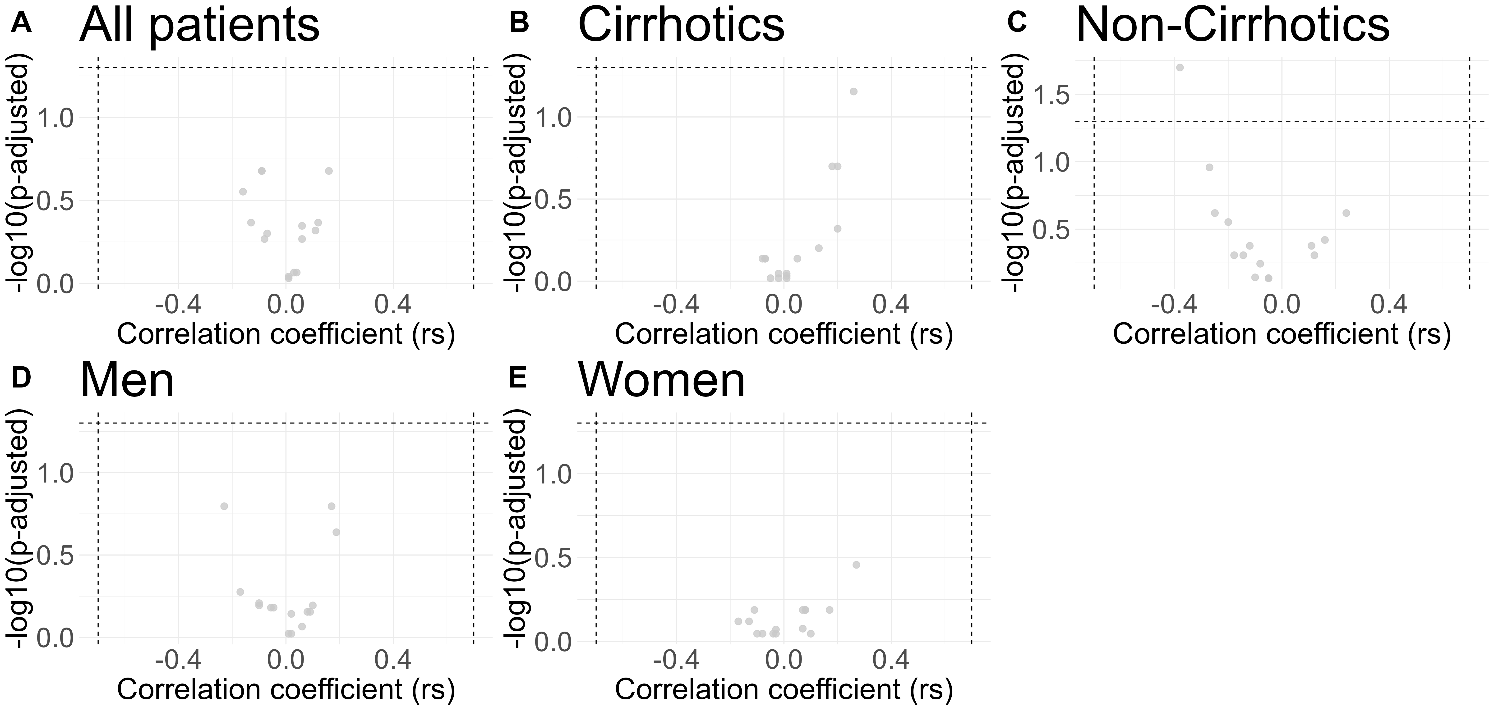


**Supplementary Figure S2.** Volcano plots of correlation coefficients (rs) and adjusted p-values between urease subunit alpha and markers for muscle function, muscle biomarkers, and serum markers.


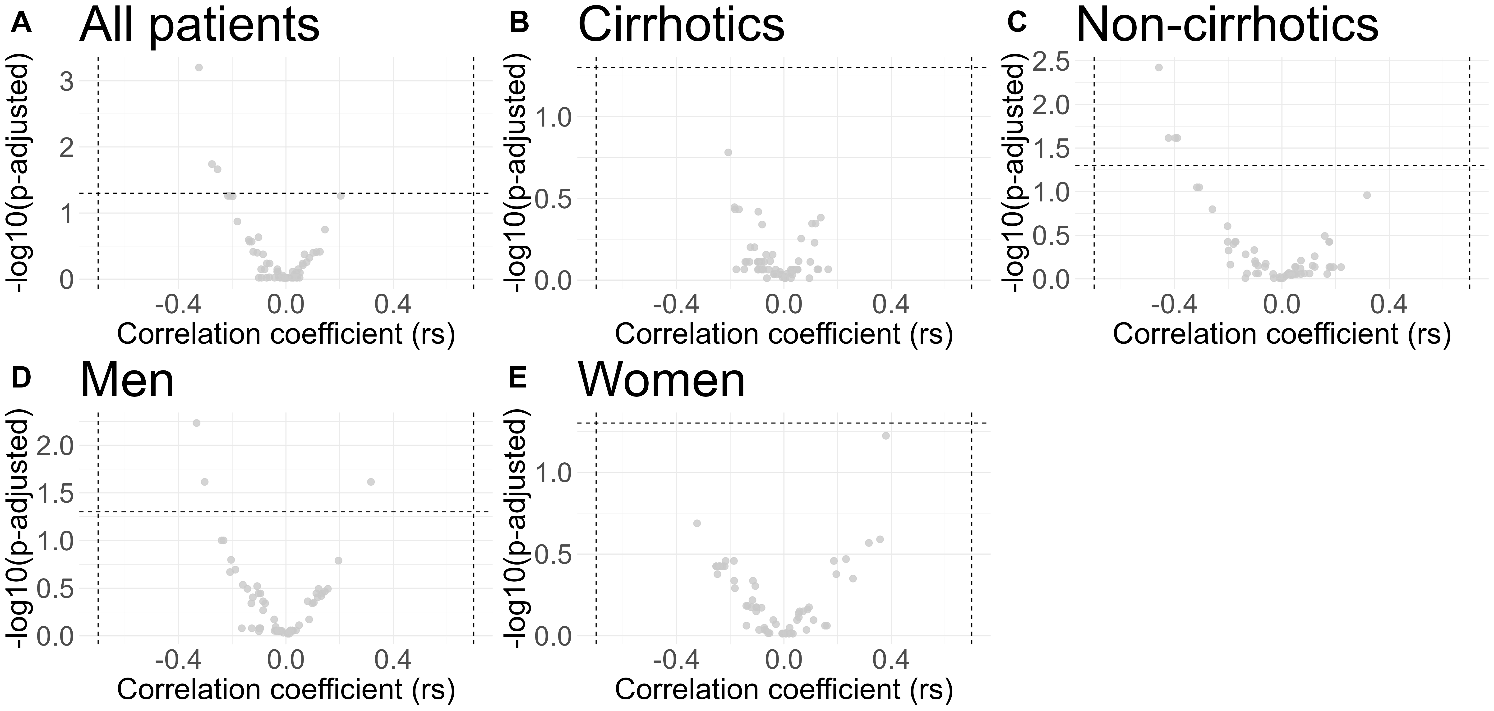


**Supplementary Figure S3.** Volcano plots of correlation coefficients (rs) and adjusted p-values between urease subunit alpha and chosen metabolites in stool and serum.


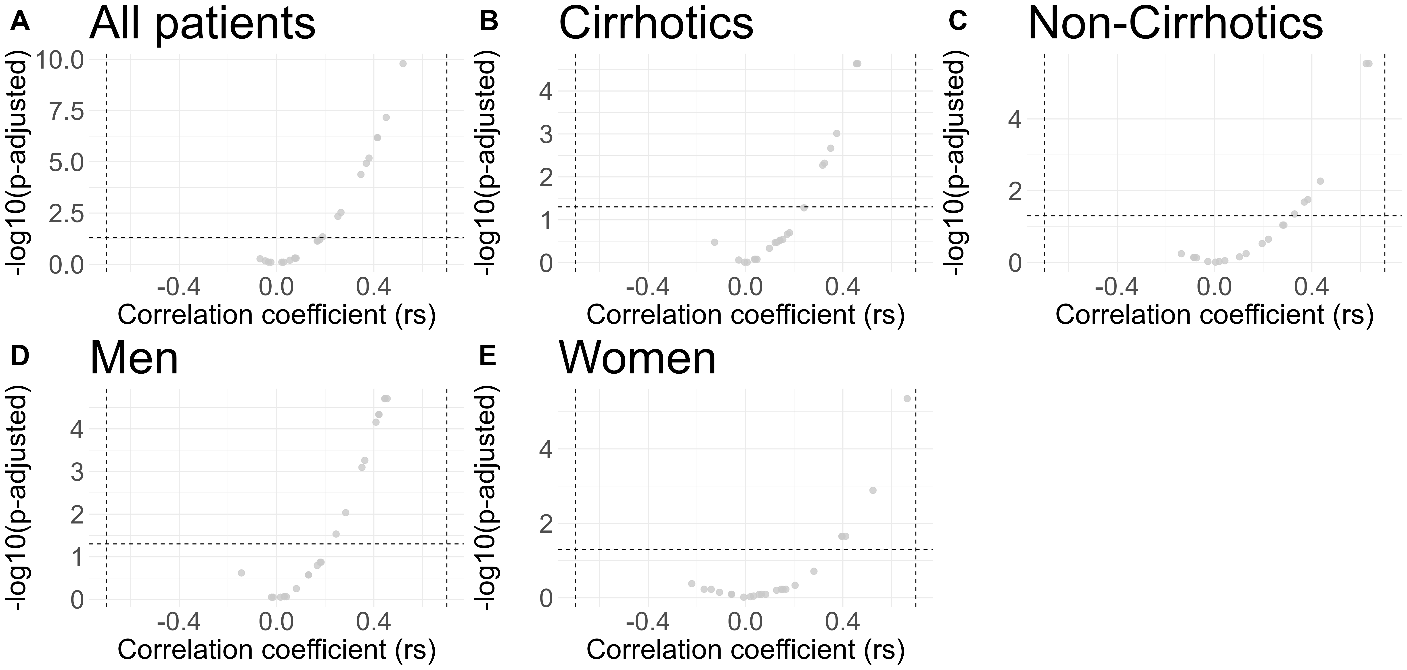


**Supplementary Figure S4.** Volcano plots of correlation coefficients (rs) and adjusted p-values between urease subunit alpha and urease-producing taxa.


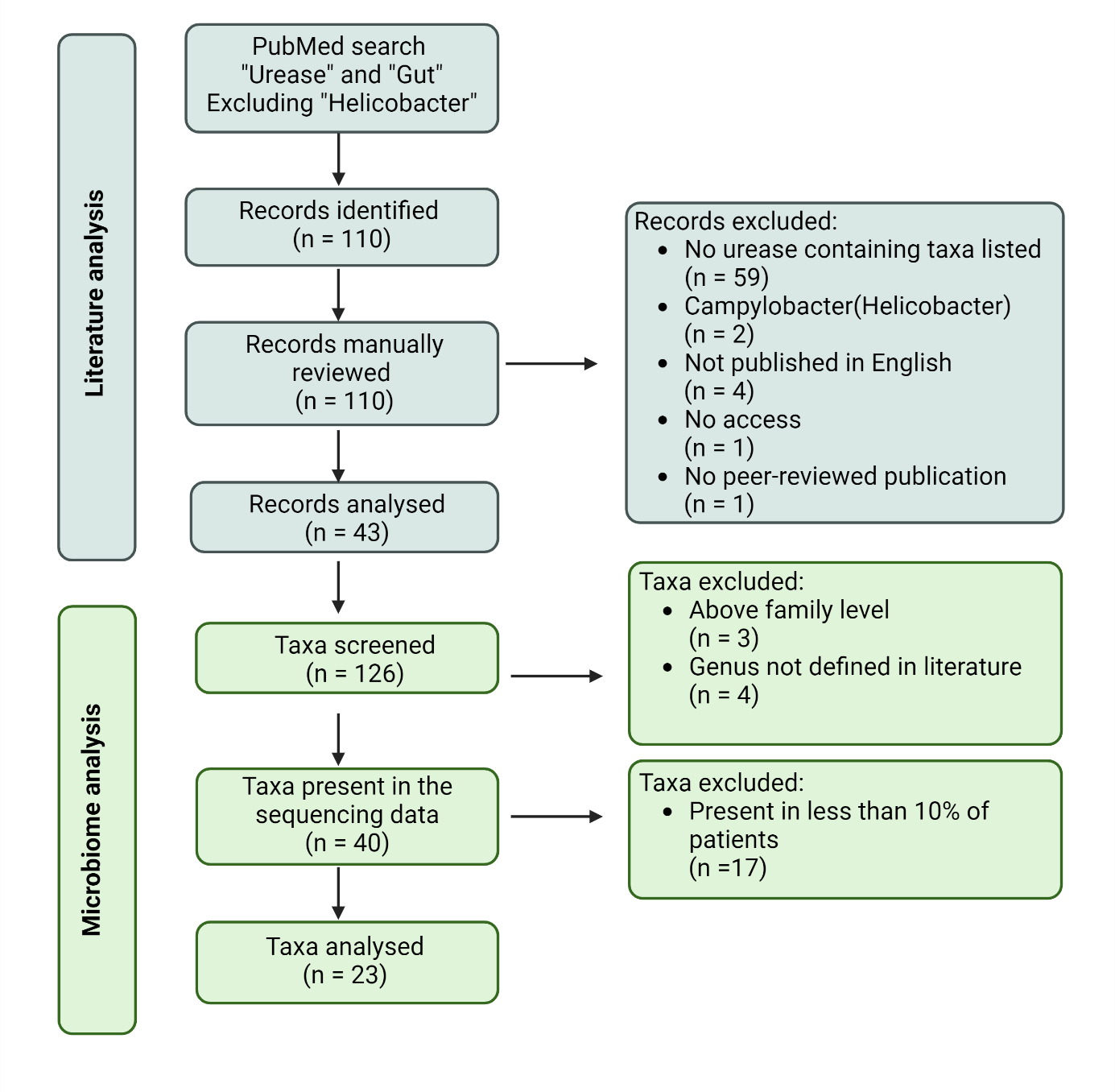


**Supplementary Figure S5.** Flow chart of the systematic literature search; PubMed was searched for urease-producing taxa and 110 records were identified. After screening 43 records were included in the analysis and 126 taxa were extracted by manually reviewing the publications. In total 40 taxa were presented in the sequencing data. 23 taxa were present in more than 10% of the patients and analyzed for their differences between patients with and without sarcopenia. Created with biorender.


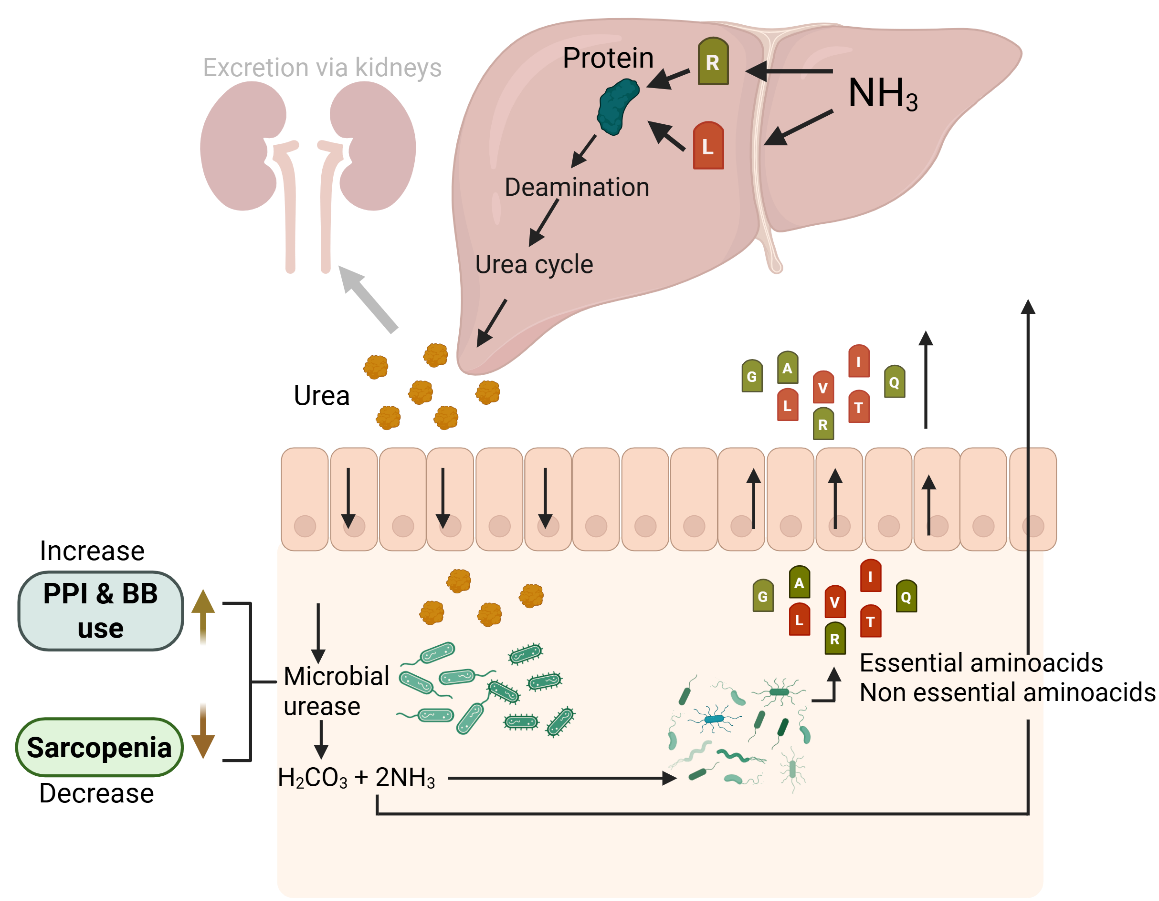


**Supplementary Figure S6.** Concept of nitrogen recycling. Proteins are broken down in the liver to urea, which is partially excreted by the kidney or into the human gut. In the gut microbial urease breaks urea down to ammonium, which then can be used for synthesis of amino acids and new proteins. Use of proton pump inhibitor (PPI) and beta-blockers (BB) increases the abundance of microbial urease, while was lower in patients with sarcopenia. Created with biorender.

# Supplementary Tables

Supplementary Table S1 Cut-offs applied for this study according to EWGSOP 2010

| **Variable** |  |  |  |  |
| --- | --- | --- | --- | --- |
|  |  |  | **BMI [kg/m²]** | **HGS [kg]** |
| **Strength** | **Hand Grip Strength (HGS) [kg]** | male | ≤ 24.0  24.1 – 26.0  26.1 – 28.0  > 28.0 | ≤ 29.0  ≤ 30.0  ≤ 30.0  ≤ 32.0 |
|  |  | female | ≤ 23.0  23.1 – 26.0  26.1 – 29.0  > 29.0 | ≤ 27.0  ≤ 17.3  ≤ 18.0  ≤ 21.0 |
| **Function** | **Gait speed [m/s]** | ≤0.8 m/s | | |
| **Mass** |  |  | **MRT/CT** | **DXA** |
|  | **Muscle Mass** | male | <52,5cm²/m² | <7 kg/m^2^ |
|  |  | female | < 38,6cm²/m | <5,5 kg/m^2^ |

**Supplementary Table S2**: Biosample IDs of SRA PRJNA933898.

*) Samples were excluded due to low sequence reads

| 33249522* | 33249485 | 33249448 | 33249411 | 33249374 |
| --- | --- | --- | --- | --- |
| 33249521* | 33249484 | 33249447 | 33249410 | 33249373 |
| 33249520* | 33249483 | 33249446 | 33249409 | 33249372 |
| 33249519 | 33249482 | 33249445 | 33249408 | 33249371 |
| 33249518* | 33249481 | 33249444 | 33249407 | 33249370 |
| 33249517 | 33249480 | 33249443 | 33249406 | 33249369 |
| 33249516 | 33249479 | 33249442 | 33249405 | 33249368 |
| 33249515 | 33249478 | 33249441 | 33249404 | 33249486 |
| 33249514 | 33249477 | 33249440 | 33249403 | 33249449 |
| 33249513 | 33249476 | 33249439 | 33249402 | 33249412 |
| 33249512 | 33249475 | 33249438 | 33249401 | 33249375 |
| 33249511 | 33249474 | 33249437 | 33249400 |  |
| 33249510 | 33249473 | 33249436 | 33249399 |  |
| 33249509 | 33249472 | 33249435 | 33249398 |  |
| 33249508 | 33249471 | 33249434 | 33249397 |  |
| 33249507 | 33249470 | 33249433 | 33249396 |  |
| 33249506 | 33249469 | 33249432 | 33249395 |  |
| 33249505 | 33249468 | 33249431 | 33249394 |  |
| 33249504 | 33249467 | 33249430 | 33249393 |  |
| 33249503 | 33249466 | 33249429 | 33249392 |  |
| 33249502 | 33249465 | 33249428 | 33249391 |  |
| 33249501 | 33249464 | 33249427 | 33249390 |  |
| 33249500 | 33249463 | 33249426 | 33249389 |  |
| 33249499 | 33249462 | 33249425 | 33249388 |  |
| 33249498 | 33249461 | 33249424 | 33249387 |  |
| 33249497 | 33249460 | 33249423 | 33249386 |  |
| 33249496 | 33249459 | 33249422 | 33249385 |  |
| 33249495 | 33249458 | 33249421 | 33249384 |  |
| 33249494 | 33249457 | 33249420 | 33249383 |  |
| 33249493 | 33249456 | 33249419 | 33249382 |  |
| 33249492 | 33249455 | 33249418 | 33249381 |  |
| 33249491 | 33249454 | 33249417 | 33249380 |  |
| 33249490 | 33249453 | 33249416 | 33249379 |  |
| 33249489 | 33249452 | 33249415 | 33249378 |  |
| 33249488 | 33249451 | 33249414 | 33249377 |  |
| 33249487 | 33249450 | 33249413 | 33249376 |  |

**Supplementary Table S3**: All measured metabolites in serum and stool

| Metabolites | |
| --- | --- |
| Stool | Serum |
| 3-4-Dihydroxybenzeneacetic acid | 2-Hydroxy-3-methylbutyric acid |
| 5-Aminopentanoic-acid | 3-Hydroxybutyric acid |
| Acetic acid | 3-Methyl-2-oxovaleric acid |
| Beta-Alanine | 4-Aminohippuric-acid |
| Betaine | Acetic acid |
| Butyric acid | Acetone |
| Choline | Citric acid |
| D-Fructose | Creatine |
| D-Galactose | D-Glucose |
| D-Glucose | D-Mannose |
| Deoxyuridine | Dimethylamine |
| Formic acid | Ethanol |
| Fumaric acid | Formic acid |
| Gallic acid | Glutamic acid |
| Glutamic acid | Glutamine |
| Glycerol | Glycine |
| Glycine | Hippuric acid |
| Histidine | Histidine |
| Hypoxanthine | Indoxyl-sulfate |
| Isoleucine | Isobutyric acid |
| Isopropyl alcohol | Isoleucine |
| L-Alanine | Ketoleucine |
| L-Asparagine | L-Alanine |
| L-Aspartic acid | L-Arginine |
| L-Tryptophan | L-Aspartic acid |
| L-Tyrosine | L-Carnitine |
| L-Valine | L-Threonine |
| Lactic acid | L-Tryptophan |
| Leucine | L-Tyrosine |
| Lysine | L-Valine |
| Malonic acid | Lactic-acid |
| Methylamine | Leucine |
| Nicotinic acid | Lysine |
| p-Cresol | Methionine |
| Phenylacetic acid | Myo-Inositol |
| Phenylalanine | Ornithine |
| Proline | Phenylalanine |
| Propionic acid | Phosphocreatine |
| Propylene glycol | Salicylic-acid |
| Sarcosine | Sebacic-acid |
| Succinic acid | Serine |
| Sucrose | Trimethylamine |
| Taurine |  |
| Trimethylamine |  |
| Uracil |  |
| Valeric acid |  |
| Xanthine |  |

Supplementary Table S4 Correlation of urease subunit alpha to markers of muscle function and muscle biomarkers. P-values (p-adj) were corrected with Benjamini-Hochberg for the groups indicated in the table.

|  | **Correlation coefficient (spearman)** | | | | | | | | | | | |
| --- | --- | --- | --- | --- | --- | --- | --- | --- | --- | --- | --- | --- |
|  | **All patients**  **(n = 152)** | | **Cirrhotic patients**  **(n = 96)** | | **Non-cirrhotic patients**  **(n = 56)** | | **Men**  **(n = 103)** | | | **Women**  **(n = 49)** | | |
| **Muscle function** | | | | | | | | | | | | |
|  | **rs** | **p-adj** | **rs** | **p-adj** | **rs** | **p-adj** | **rs** | **p-adj** | | **rs** | **p-adj** | |
| **Muscle mass** | 0.12 | 0.43 | 0.26 | 0.07 | -0.10 | 0.72 | 0.10 | 0.64 | | 0.27 | | 0.35 |
| **Handgrip strength** | -0.13 | 0.43 | -0.07 | 0.73 | -0.20 | 0.28 | -0.17 | 0.53 | | 0.08 | | 0.65 |
| **Mid-arm muscle circumference** | 0.01 | 0.91 | 0.05 | 0.73 | -0.05 | 0.73 | 0.01 | 0.95 | | 0.08 | | 0.65 |
| **Triceps skinfold thickness** | -0.08 | 0.54 | 0.01 | 0.93 | -0.25 | 0.24 | -0.10 | 0.64 | | -0.11 | | 0.65 |
| **Gait speed** | 0.06 | 0.54 | 0.13 | 0.63 | -0.05 | 0.73 | 0.02 | 0.95 | | 0.17 | | 0.65 |
| **Chair raise** | 0.06 | 0.45 | -0.07 | 0.73 | 0.24 | 0.24 | 0.06 | 0.86 | | 0.07 | | 0.65 |
| **Muscle biomarker** | | | | | | | | | | | | |
| **Myostatin** | -0.07 | 0.50 | -0.05 | 0.96 | -0.081 | 0.570 | -0.046 | | 0.66 | -0.03 | | 0.85 |
| **Irisin** | -0.09 | 0.21 | -0.02 | 0.96 | -0.178 | 0.493 | -0.056 | | 0.66 | -0.17 | | 0.76 |
| **FGF-21** | 0.16 | 0.21 | 0.20 | 0.48 | 0.121 | 0.493 | 0.188 | | 0.23 | 0.07 | | 0.84 |
| **IGF-1** | -0.09 | 0.21 | 0.01 | 0.96 | -0.144 | 0.493 | -0.100 | | 0.62 | -0.13 | | 0.76 |
| **Serum marker** | | | | | | | | | | | | |
| **Total Protein** | 0.03 | 0.86 | 0.18 | 0.20 | -0.27 | 0.11 | 0.08 | 0.70 | | -0.03 | | 0.9 |
| **Albumin** | -0.16 | 0.28 | 0.01 | 0.90 | -0.38 | 0.02 | -0.23 | 0.16 | | -0.10 | | 0.9 |
| **Urea** | 0.01 | 0.93 | -0.08 | 0.73 | 0.16 | 0.38 | 0.09 | 0.70 | | -0.08 | | 0.9 |
| **Potassium** | 0.11 | 0.48 | 0.20 | 0.20 | -0.12 | 0.42 | 0.17 | 0.16 | | -0.04 | | 0.9 |
| **CRP** | 0.04 | 0.86 | -0.02 | 0.90 | 0.11 | 0.42 | 0.02 | 0.72 | | 0.1 | | 0.9 |

Supplementary Table S5 Correlation of urease subunit alpha of chosen metabolites in stool and serum. P-values (p-adj) were corrected with Benjamini-Hochberg for the groups indicated in the table.

|  | **Correlation coefficient (spearman)** | | | | | | | | | | | | | |
| --- | --- | --- | --- | --- | --- | --- | --- | --- | --- | --- | --- | --- | --- | --- |
|  | **All patients**  **(n = 138)** | | | **Cirrhotic patients**  **(n = 86)** | | **Non-cirrhotic patients**  **(n = 52)** | | | **Men**  **(n = 96)** | | | **Women**  **(n = 43)** | | |
| **Stool metabolites** | | | | | | | | | | | | | | |
|  | **rs** | **p-adj** | | **rs** | **p-adj** | **rs** | **p-adj** | | **rs** | **p-adj** | | **rs** | **p-adj** | |
| **Short Chain Fatty Acids** | | | | | | | | | | | | | | |
| Acetic acid | -0.01 | | 0.88 | 0.1 | 0.97 | -0.20 | | 0.25 | 0.02 | | 0.88 | -0.10 | 0.71 | |
| Butyric acid | 0.13 | | 0.39 | 0.03 | 0.97 | 0.32 | | 0.11 | 0.02 | | 0.88 | 0.38 | 0.06 | |
| Formic acid | -0.12 | | 0.39 | -0.06 | 0.97 | -0.26 | | 0.16 | -0.21 | | 0.21 | 0.07 | 0.71 | |
| Propionic acid | 0.05 | | 0.80 | 0.01 | 0.97 | 0.071 | | 0.62 | 0.04 | | 0.88 | 0.06 | 0.71 | |
| Valeric acid | 0.04 | | 0.80 | 0.004 | 0.97 | 0.16 | | 0.32 | -0.04 | | 0.88 | 0.23 | 0.34 | |
| **Proteinogenic Amino acids** | | | | | | | | | | | | | | |
| Glutamic acid | -0.04 | | 0.95 | -0.09 | 0.77 | -0.03 | | 0.88 | -0.02 | | 0.89 | -0.07 | | 0,92 |
| Glycine | 0.05 | | 0.95 | -0.01 | 0.92 | 0.18 | | 0.73 | -0.10 | | 0.83 | 0.36 | | 0,26 |
| Histidine | -0.01 | | 0.95 | 0.03 | 0.92 | -0.10 | | 0.87 | -0.10 | | 0.83 | 0.15 | | 0,87 |
| Isoleucine | -0.01 | | 0.95 | -0.13 | 0.77 | 0.19 | | 0.73 | -0.03 | | 0.89 | -0.01 | | 0,97 |
| L-Alanine | -0.02 | | 0.95 | -0.10 | 0.77 | 0.04 | | 0.88 | 0.02 | | 0.89 | -0.09 | | 0,92 |
| L-Asparagine | 0.04 | | 0.95 | -0.01 | 0.92 | 0.08 | | 0.87 | 0.03 | | 0.89 | 0.03 | | 0,97 |
| L-Aspartic acid | 0.02 | | 0.95 | -0.08 | 0.77 | 0.18 | | 0.73 | 0.02 | | 0.89 | 0.02 | | 0,97 |
| Leucine | -0.10 | | 0.95 | -0.14 | 0.77 | 0.01 | | 0.93 | -0.16 | | 0.83 | 0.01 | | 0,97 |
| Lysine | -0.07 | | 0.95 | -0.1 | 0.77 | -0.10 | | 0.87 | -0.03 | | 0.89 | -0.14 | | 0,87 |
| L-Tyrosine | -0.09 | | 0.95 | -0.14 | 0.77 | 0.06 | | 0.88 | -0.10 | | 0.83 | -0.07 | | 0,92 |
| L-Tryptophan | 0.03 | | 0.95 | -0.03 | 0.92 | 0.10 | | 0.87 | -0.10 | | 0.83 | 0.26 | | 0,45 |
| L-Valin | 0.01 | | 0.95 | -0.04 | 0.92 | 0.05 | | 0.88 | -0.04 | | 0.89 | 0.08 | | 0,92 |
| Phenylalanine | 0.03 | | 0.95 | -0.03 | 0.77 | 0.22 | | 0.73 | -0.13 | | 0.83 | 0.32 | | 0,27 |
| Proline | 0.04 | | 0.95 | 0.10 | 0.77 | -0.13 | | 0.87 | -0.02 | | 0.89 | 0.16 | | 0,87 |
| **Amino acid metabolism** | | | | | | | | | | | | | | |
| 5-Aminopentanoic acid | -0.01 | | 0.94 | -0.11 | 0.63 | 0.17 | | 0.88 | 0.01 | | 0.89 | -0.06 | | 0.96 |
| beta-Alanine | -0.01 | | 0.94 | -0.04 | 0.70 | 0.03 | | 0.88 | -0.02 | | 0.89 | 0.01 | | 0.96 |
| p-Cresole | -0.06 | | 0.94 | -0.13 | 0.63 | 0.07 | | 0.88 | -0.10 | | 0.89 | 0.03 | | 0.96 |
| Sarcosine | -0.03 | | 0.94 | -0.07 | 0.70 | 0.02 | | 0.88 | -0.03 | | 0.89 | -0.05 | | 0.96 |
| **Muscle Heath** | | | | | | | | | | | | | | |
| Lactic acid | -0.10 | | 0.23 | -0.10 | 0.38 | -0.10 | | 0.47 | -0.11 | | 0.30 | -0.11 | | 0.50 |
| **Vitamins** | | | | | | | | | | | | | | |
| Choline | 0.069 | | 0.42 | 0.14 | 0.41 | -0.06 | | 0.67 | 0.08 | | 0.43 | 0.02 | | 0.89 |
| Nicotinic acid | -0.085 | | 0.42 | -0.08 | 0.46 | -0.10 | | 0.67 | -0.09 | | 0.43 | -0.07 | | 0.89 |
| **Serum metabolites** | | | | | | | | | | | | | | |
| **Short Chain Fatty Acids** | | | | | | | | | | | | | | |
| Acetic acid | -0.03 | | 0.71 | -0.04 | 0.92 | -0.03 | | 0.98 | -0.08 | | 0.46 | 0.11 | | 0.80 |
| Isobutyric acid | -0.09 | | 0.71 | -0.21 | 0.92 | -0.14 | | 0.98 | -0.13 | | 0.46 | 0.05 | | 0.80 |
| Formic acid | 0.05 | | 0.71 | 0.01 | 0.17 | 0.003 | | 0.98 | 0.10 | | 0.46 | -0.04 | | 0.80 |
| **Proteinogenic Amino acids** | | | | | | | | | | | | | | |
| Glutamic acid | 0.204 | | 0.05 | 0.13 | 0.86 | 0.18 | | 0.38 | 0.32 | | 0.02 | -0.03 | | 0.85 |
| Glutamine | -0.107 | | 0.40 | 0.03 | 0.86 | -0.20 | | 0.38 | -0.12 | | 0.39 | -0.08 | | 0.67 |
| Glycine | 0.114 | | 0.39 | 0.13 | 0.86 | 0.18 | | 0.38 | 0.15 | | 0.34 | 0.09 | | 0.67 |
| Histidine | -0.006 | | 0.95 | 0.05 | 0.86 | -0.10 | | 0.63 | 0.10 | | 0.45 | -0.25 | | 0.38 |
| Isoleucine | -0.198 | | 0.06 | -0.03 | 0.86 | -0.39 | | 0.02 | -0.16 | | 0.29 | -0.24 | | 0.38 |
| L-Alanine | -0.061 | | 0,58 | 0.05 | 0.86 | -0.17 | | 0.38 | 0.01 | | 0.95 | -0.23 | | 0.38 |
| L-Arginine | -0.26 | | 0.02 | -0.06 | 0.86 | -0.42 | | 0.02 | -0.23 | | 0.10 | -0.25 | | 0.38 |
| L-Aspartic acid | 0.06 | | 0.58 | 0.17 | 0.86 | 0.04 | | 0.91 | 0.13 | | 0.38 | -0.10 | | 0.67 |
| Leucine | -0.28 | | 0.02 | -0.18 | 0.86 | -0.40 | | 0.02 | -0.30 | | 0.02 | -0.22 | | 0.38 |
| L-Tryptophan | -0.14 | | 0.25 | -0.08 | 0.86 | -0.17 | | 0.38 | -0.09 | | 0.54 | -0.24 | | 0.38 |
| L-Threonine | -0.07 | | 0.58 | -0.10 | 0.86 | -0.003 | | 0.98 | -0.04 | | 0.81 | -0.14 | | 0.66 |
| L-Tyrosine | -0.03 | | 0.77 | -0.15 | 0.86 | -0.14 | | 0.52 | 0.007 | | 0.95 | -0.10 | | 0.67 |
| L-Valine | -0.22 | | 0.05 | -0.10 | 0.86 | -0.31 | | 0.09 | -0.20 | | 0.16 | -0.24 | | 0.38 |
| Lysin | -0.21 | | 0.05 | -0.08 | 0.86 | -0.32 | | 0.09 | -0.24 | | 0.10 | -0.14 | | 0.66 |
| Methionine | 0.09 | | 0.48 | 0.01 | 0.86 | 0.07 | | 0.76 | 0.12 | | 0.39 | 0.06 | | 0.77 |
| Phenylalanine | 0.10 | | 0.40 | 0.03 | 0.86 | 0.12 | | 0.55 | 0.20 | | 0.16 | -0.12 | | 0.67 |
| Serine | -0.03 | | 0.77 | 0.03 | 0.86 | -0.01 | | 0.98 | 0.05 | | 0.77 | -0.18 | | 0.51 |
| **Non-Proteinogenic Amino acids** | | | | | | | | | | | | | | |
| L-Carnitine | -0.08 | | 0.71 | 0.03 | 0.89 | -0.18 | | 0.40 | -0.04 | | 0.67 | -0.19 | | 0.46 |
| Ornithine | 0.03 | | 0.77 | -0.02 | 0.89 | -0.01 | | 0.95 | 0.09 | | 0.67 | -0.12 | | 0.46 |
| **Amino acid metabolism** | | | | | | | | | | | | | | |
| Hippuric acid | 0.08 | | 0.55 | 0.11 | 0.59 | 0.05 | | 0.73 | 0.11 | | 0.36 | -0.01 | | 0.97 |
| 3-Hydroxybutyric acid | -0.13 | | 0.27 | -0.17 | 0.37 | -0.06 | | 0.73 | -0.19 | | 0.20 | 0.09 | | 0.69 |
| Indoxyl sulfate | 0.03 | | 0.83 | 0.05 | 0.77 | 0.05 | | 0.73 | 0.14 | | 0.36 | -0.22 | | 0.35 |
| Ketoleucine | -0.32 | | 0.001 | -0.18 | 0.37 | -0.46 | | 0.004 | -0.33 | | 0.01 | -0.32 | | 0.21 |
| 3-Methyl-2-oxovaleric acid | -0.004 | | 0.97 | -0.01 | 0.96 | 0.12 | | 0.73 | -0.10 | | 0.36 | 0.19 | | 0.35 |
| Salicylic acid | -0.14 | | 0.27 | -0.05 | 0.77 | -0.20 | | 0.47 | -0.10 | | 0.36 | -0.19 | | 0.35 |
| **Muscle Health** | | | | | | | | | | | | | | |
| Acetone | 0.15 | | 0.18 | 0.11 | 0.45 | 0.11 | | 0.71 | 0.12 | | 0.32 | 0.20 | | 0.42 |
| Creatine | 0.06 | | 0.62 | 0.12 | 0.45 | 0.03 | | 0.86 | 0.16 | | 0.32 | -0.12 | | 0.60 |
| Lactic acid | 0.01 | | 0.95 | 0.06 | 0.56 | -0.19 | | 0.69 | -0.01 | | 0.93 | 0.06 | | 0.73 |
| Phosphocreatine | -0.18 | | 0.13 | -0.18 | 0.36 | -0.09 | | 0.71 | -0.14 | | 0.32 | -0.25 | | 0.42 |

Supplementary Table S6 Taxa identified in the systematic literature search, including references.

| \| ***Taxa*** \| ***Reference*** \| \| --- \| --- \| \| **Phylum *Actinomycetota:*** \|  \| \| *Actinomyces naeslundii* \| (Bajaj et al., 2021) \| \| *Bifidobacterium longum subspecies longum, B. infantis, B.infantis subsp. infantis ATCC 15702, B. suis, B. subtile, B. kashiwanohense, B. scardovii, B. bifidum* \| (Suzuki et al., 1979; Yuan et al., 2020; Schimmel et al., 2021) \| \| *Eggerthella sp.YY7918* \| (Xu et al., 2020) \| \| *Eggerthella lenta ATCC 43055* \| (Yuan et al., 2020) \| \| *Cellulomonadaceae* \| (Lau et al., 2021) \| \| *Micrococcaceae* \| (Lau et al., 2021) \| \| *Dermabacteraceae* \| (Lau et al., 2021) \| \| **Phylum *Ascomycota:*** \|  \| \| *Fusarium solani (ATCC MYA 4552)* \| (Scully et al., 2012) \| \| **Phylum *Bacillota:*** \|  \| \| *Clostridia, Uncl. Clostridiales, Clostridiaceae, Clostridium perfringens, Clostridium symbiosum (APhL), Clostridium spp., Clostridia (ASF492), Clostridium perfringens ATCC 13124, Clostridium difficile ATCC 700057* \| (Suzuki et al., 1979; Shen et al., 2015; Inoue et al., 2018; Xu et al., 2020; Yuan et al., 2020; Lau et al., 2021; Ryvchin et al., 2021; Xie et al., 2021; Regan et al., 2022; Zhao et al., 2022) \| \| *Eubacterium, Eubacterium aerofaciens, E. lentum* \| (Suzuki et al., 1979; Zhao et al., 2022) \| \| *Blautia* \| (Ryvchin et al., 2021) \| \| *Uncl. Ruminococcaceae, Ruminococcus, Ruminococcus bicirculans, strain 80/3* \| (Wegmann et al., 2014; Inoue et al., 2018; Ryvchin et al., 2021; Zhao et al., 2022) \| \| *Streptococcus, S. thermophilus, Streptococcus salivarius, Streptococcus vestibularis* \| (Arvola et al., 1999; Rai et al., 2015; Uriot et al., 2016; Inoue et al., 2018; Bajaj et al., 2021; Ryvchin et al., 2021; Schimmel et al., 2021; Yukawa-Muto et al., 2022) \| \| *Peptococcus asaccharolyticus, P. prevotii* \| (Suzuki et al., 1979) \| \| *Peptostreptococcus productus, P. prevoti ATCC 9321* \| (Suzuki et al., 1979; Yuan et al., 2020) \| \| *Enterococcus faecium* \| (Teh et al., 2021) \| \| *Lactobacillus (ASF361), L. fermentum, L. reuteri 100-23, L. rhamnosus ATCC 53103, L. casei ATCC 393, L. plantarum* \| (Suzuki et al., 1979; Wilson et al., 2012, 2014; Shen et al., 2015; Regan et al., 2022; Wang et al., 2022) \| \| *Coprococcus catus* \| (Xu et al., 2020) \| \| *Sporosarcina pasteurii 6452* \| (Ranganathan et al., 2006) \| \| *Paenibacillus sp. strain P118* \| (de Castro et al., 2011) \| \| *Roseburia spp.* \| (Xie et al., 2021) \| \| *Lachnoclostridium spp.* \| (Xie et al., 2021) \| \| *Priestia aryabhattai* \| (Lailaja et al., 2022) \| \| *Bacillus altitudinis* \| (Lailaja et al., 2022) \| \| **Phylum *Bacteroidota:*** \|  \| \| *Bacteroidales, Bacteroides fragilis, Bacteroides multiacidus, Bacteroides bifidum ATCC 1569* \| (Xu et al., 2020; Ryvchin et al., 2021) \| \| *Odoribacter splanchnicus* \| (Zhang et al., 2013) \| \| *Alistipes* \| (Regan et al., 2022) \| \| **Phylum *Euryarchaeota*:** \|  \| \| *Thermoplasma acidophilum* \| (Xu et al., 2020) \| \| **Phylum *Fibrobacterota:*** \|  \| \| *Fibrobacter, F. succinogenes UWEL, UWR1, UWR 4, UWT1, UWH9, UWH1, UWH5, UWH8, UWH3, UWH6, UWB7, UWB10* \| (Neumann and Suen, 2018) \| \| **Phylum *Fusobacteriota*** \|  \| \| *Fusobacterium necrophorum, Fusobacterium necrophorum ATCC 25286, F.varium* \| (Suzuki et al., 1979; Yuan et al., 2020) \| \| **Phylum *Proteobacteria:*** \| (Ryvchin et al., 2021) \| \| *Enterobacteriaceae, Enterobacter, E. cloacae, Enterobacter sp. OLF* \| (Rai et al., 2015; Xu et al., 2020; Lau et al., 2021; Schimmel et al., 2021) \| \| *Salmonella, Salmonella spp ATCC 35987* \| (Ramatla et al., 2020; Yuan et al., 2020) \| \| *Yersinia enterocolitica ATCC 23715* \| (Yuan et al., 2020) \| \| *Klebsiella spp., Klebsiella pneumoniae, K. oxytoca, Klebsiella aerogenes, Klebsiella pneumoniae ATCC 700603* \| (Zhang et al., 2013, 2020; Yuan et al., 2020; Schimmel et al., 2021; Teh et al., 2021; Ren et al., 2022) \| \| *Escherichia, Escherichia fergusonii* \| (Hu et al., 2020; Schimmel et al., 2021) \| \| *Pseudomonadales, Pseudomonadaceae, Pseudomonas.* \| (Hu et al., 2018; Inoue et al., 2018; Lau et al., 2021) \| \| *Proteus spp., P. mirabilis, P. vulgaris, P. penneri* \| (Zhang et al., 2013; Hamilton et al., 2018; Yuan et al., 2020; Teh et al., 2021) \| \| *Vibrio parahaemolyticus* \| (Kongrueng et al., 2018) \| \| *Serratia marcescens SM03* \| (Srinivasan and Rajamohan, 2019) \| \| *Desulfomicrobium baculatum* \| (Xu et al., 2020) \| \| *Desulfovibrio diazotrophicus sp. nov., Desulfovibrio desulfuricans strain AY5* \| (Karnachuk et al., 2021; Sayavedra et al., 2021) \| \| *Rickettsia prowazekii* \| (Xu et al., 2020) \| \| *Unclassified Moraxellaceae, Moraxellaceae* \| (Inoue et al., 2018; Lau et al., 2021) \| \| *Morganella morganii* \| (Teh et al., 2021; Ren et al., 2022) \| \| *Alcaligeneceae* \| (Rai et al., 2015) \| \| *Brucella pseudogrignonense, B. abortus, B. anthropi ATCC 49188, B. anthropi W13P3, B. intermedia LMG 3301, Brucella melitensis* \| (Paixão et al., 2009; Zhukova et al., 2022) \| \| *Bradyrhizobium japonicum, B. neotropicale* \| (Zhukova et al., 2022) \| \| *Agrobacterium rhizogenes, A. tumefaciens* \| (Zhukova et al., 2022) \| \| *Rhizobiales, Rhizobium leguminosarum* \| (Hu et al., 2018; Zhukova et al., 2022) \| \| *Ensifer adhaerens* \| (Zhukova et al., 2022) \| \| *Hoeflea phototrophica, H. olei* \| (Zhukova et al., 2022) \| \| *Bosea vaviloviae, B. thiooxidans* \| (Zhukova et al., 2022) \| \| *Mezorhizobium amorphae, M. alhagi* \| (Zhukova et al., 2022) \| \| *Ca. Tokpelaia hoelldoblerii, Candidatus Tokpelaia hoelldoblerii strain Hsal, Candidatus Erwinia dacicola* \| (Neuvonen et al., 2016; Blow et al., 2020; Zhukova et al., 2022) \| \| *Bartonella apis, Bartonella apis sp. nov.* \| (Kešnerová et al., 2016; Zhukova et al., 2022) \| \| *Alteromonadaceae* \| (Lau et al., 2021) \| \| *Halomonadaceae* \| (Lau et al., 2021) \| \| *Methylococcaceae* \| (Lau et al., 2021) \| \| *Polyangiaceae* \| (Lau et al., 2021) \| \| *Xanthomonadaceae* \| (Lau et al., 2021) \| \| **Phylum *Spirochaetes:*** \|  \| \| *Turneriella parva* \| (Xu et al., 2020) \| \| **Phylum *Zygomycota:*** \|  \| \| *Gilbertella persicaria* \| (Huët et al., 2020) \| |
| --- | --- | --- | --- | --- | --- | --- | --- | --- | --- | --- | --- | --- | --- | --- | --- | --- | --- | --- | --- | --- | --- | --- | --- | --- | --- | --- | --- | --- | --- | --- | --- | --- | --- | --- | --- | --- | --- | --- | --- | --- | --- | --- | --- | --- | --- | --- | --- | --- | --- | --- | --- | --- | --- | --- | --- | --- | --- | --- | --- | --- | --- | --- | --- | --- | --- | --- | --- | --- | --- | --- | --- | --- | --- | --- | --- | --- | --- | --- | --- | --- | --- | --- | --- | --- | --- | --- | --- | --- | --- | --- | --- | --- | --- | --- | --- | --- | --- | --- | --- | --- | --- | --- | --- | --- | --- | --- | --- | --- | --- | --- | --- | --- | --- | --- | --- | --- | --- | --- | --- | --- | --- | --- | --- | --- | --- | --- | --- | --- | --- | --- | --- | --- | --- | --- | --- | --- | --- | --- | --- | --- | --- | --- | --- | --- | --- | --- |

Supplementary Table S7 Mean and standard deviation of extracted taxa in patients with and without sarcopenia for the analyzed groups (all patients, cirrhosis state, sex). P-values were adjusted (p-adj) for each group.

| -) Taxa was present in less than 10% of patients and excluded from further analysis,  *) only genus/family was used for the calculation of all taxa | | | | | |
| --- | --- | --- | --- | --- | --- |
|  | **Mean ± Standard Deviation** | | |  |  |
| **Taxa** | **All patients (n = 152)** | | |  |  |
|  | **Sarcopenia** | **No Sarcopenia** | **p-adj** |  |  |
| *Alistipes* | 0.092 ± 0.081 | 0.084 ± 0.066 | 0.935 |  |  |
| *Bacteroides fragilis* | 0.023 ± 0.041 | 0.013 ± 0.030 | 0.238 |  |  |
| *Bifidobacterium bifidum* | - | - | - |  |  |
| *Bifidobacterium longum subsp. longum* | - | - | - |  |  |
| *Bifidobacterium scardovii* | - | - | - |  |  |
| *Blautia* | 0.011 ± 0.013 | 0.010 ± 0.012 | 0.935 |  |  |
| *Clostridiaceae 1* | 0.007 ± 0.045 | 0.005 ± 0.016 | 0.238 |  |  |
| *Clostridioides difficile* | - | - | - |  |  |
| *Clostridium perfringens* | - | - | - |  |  |
| *Dermabacteraceae* | - | - | - |  |  |
| *Desulfovibrio desulfuricans* | - | - | - |  |  |
| *Eggerthella* | 0.000 ± 0.001 | 0.000 ± 0.000 | 0.930 |  |  |
| *Eggerthella YY918** | - | - | - |  |  |
| *Enterobacter** | 0.001 ± 0.008 | 0.003 ± 0.017 | 0.930 |  |  |
| *Enterobacteriaceae* | 0.067 ± 0.098 | 0.058 ± 0.068 | 0.930 |  |  |
| *Enterococcus faecium* | 0.008 ± 0.067 | 0.021 ± 0.114 | 0.930 |  |  |
| *Escherichia Shigella** | 0.049 ± 0.083 | 0.040 ± 0.056 | 0.930 |  |  |
| *Klebsiella* | 0.010 ± 0.046 | 0.010 ± 0.033 | 0.930 |  |  |
| *Klebsiella pneumoniae** | 0.010 ± 0.046 | 0.009 ± 0.031 | 0.930 |  |  |
| *Lachnoclostridium* | 0.007 ± 0.016 | 0.006 ± 0.010 | 0.930 |  |  |
| *Lactobacillus* | 0.022 ± 0.066 | 0.011 ± 0.038 | 0.930 |  |  |
| *Lactobacillus fermentum** | 0.001 ± 0.007 | 0.001 ± 0.002 | 0.935 |  |  |
| *Lactobacillus plantarum** | - | - | - |  |  |
| *Lactobacillus reuteri** | - | - | - |  |  |
| *Lactobacillus rhamnosus** | 0.000 ± 0.001 | 0.000 ± 0.002 | 0.431 |  |  |
| *Micrococcaceae* | 0.000 ± 0.000 | 0.000 ± 0.000 | 0.930 |  |  |
| *Moraxellaceae* | 0.000 ± 0.000 | 0.000 ± 0.001 | 0.930 |  |  |
| *Morganella morganii* | - | - | - |  |  |
| *Polyangiaceae* | - | - | - |  |  |
| *Proteus* | - | - | - |  |  |
| *Proteus vulgaris* | - | - | - |  |  |
| *Pseudomonadaceae* | 0.001 ± 0.002 | 0.000 ± 0.000 | 0.431 |  |  |
| *Pseudomonas** | 0.001 ± 0.002 | 0.000 ± 0.000 | 0.431 |  |  |
| *Roseburia* | 0.018 ± 0.026 | 0.012 ± 0.018 | 0.542 |  |  |
| *Ruminococcaceae* | 0.121 ± 0.088 | 0.129 ± 0.080 | 0.930 |  |  |
| *Streptococcus* | 0.010 ± 0.017 | 0.017 ± 0.034 | 0.930 |  |  |
| *Streptococcus sp.** | 0.008 ± 0.014 | 0.014 ± 0.031 | 0.542 |  |  |
| *Salmonella* | - | - | - |  |  |
| *Salmonella enterica** | - | - | - |  |  |
| *Xanthomonadaceae* | - | - | - |  |  |
| All Taxa | 0.397 ± 0.164 | 0.376 ± 0.153 | 0.930 |  |  |

|  | **Mean ± Standard Deviation** | | | | | |
| --- | --- | --- | --- | --- | --- | --- |
| **Taxa** | **Cirrhotic patients**  **(n = 96)** | | | **Non-cirrhotic patients**  **(n = 56)** | | |
|  | **Sarcopenia** | **No Sarcopenia** | **p-adj** | **Sarcopenia** | **No Sarcopenia** | **p-adj** |
| *Alistipes* | 0.090 ± 0.088 | 0.073 ± 0.060 | 0.869 | 0.094 ± 0.069 | 0.103 ± 0.007 | 0.827 |
| *Bacteroides fragilis* | 0.025 ± 0.043 | 0.016 ± 0.037 | 0.295 | 0.019 ± 0.038 | 0.007 ± 0.003 | 0.827 |
| *Bifidobacterium bifidum* | - | - | - | - | - | - |
| *Bifidobacterium longum subsp. longum* | - | - | - | - | - | - |
| *Bifidobacterium scardovii* | - | - | - | - | - | - |
| *Blautia* | 0.011 ± 0.013 | 0.012 ± 0.013 | 0.869 | 0.012 ± 0.012 | 0.008 ± 0.013 | 0.827 |
| *Clostridiaceae 1* | 0.004 ± 0.022 | 0.006 ± 0.020 | 0.607 | 0.012 ± 0.069 | 0.003 ± 0.020 | 0.398 |
| *Clostridioides difficile* | - | - | - | - | - | - |
| *Clostridium perfringens* | - | - | - | - | - | - |
| *Dermabacteraceae* | - | - | - | - | - | - |
| *Desulfovibrio desulfuricans* | - | - | - | - | - | - |
| *Eggerthella* | 0.000 ± 0.001 | 0.000 ± 0.000 | 0.884 | 0.000 ± 0.000 | 0.000 ± 0.061 | 0.973 |
| *Eggerthella YY918** | - | - | - | - | - | - |
| *Enterobacter** | - | - | - | 0.002 ± 0.011 | 0.000 ± 0.073 | 0.973 |
| *Enterobacteriaceae* | 0.069 ± 0.101 | 0.069 ± 0.073 | 0.835 | 0.063 ± 0.093 | 0.040 ± 0.060 | 0.845 |
| *Enterococcus faecium* | 0.013 ± 0.083 | 0.033 ± 0.143 | 0.942 | 0.000 ± 0.002 | 0.000 ± 0.143 | 0.827 |
| *Escherichia Shigella** | 0.057 ± 0.095 | 0.045 ± 0.061 | 0.945 | 0.037 ± 0.053 | 0.030 ± 0.000 | 0.827 |
| *Klebsiella* | 0.005 ± 0.024 | 0.011 ± 0.034 | 0.650 | 0.020 ± 0.069 | 0.008 ± 0.034 | 0.827 |
| *Klebsiella pneumoniae** | 0.005 ± 0.024 | 0.010 ± 0.033 | 0.650 | 0.019 ± 0.068 | 0.008 ± 0.034 | 0.827 |
| *Lachnoclostridium* | 0.007 ± 0.017 | 0.004 ± 0.007 | 0.523 | 0.007 ± 0.014 | 0.009 ± 0.015 | 0.827 |
| *Lactobacillus* | 0.020 ± 0.052 | 0.017 ± 0.048 | 0.945 | 0.026 ± 0.086 | 0.001 ± 0.048 | 0.827 |
| *Lactobacillus fermentum** | 0.002 ± 0.008 | 0.001 ± 0.003 | 0.747 | - | - | - |
| *Lactobacillus plantarum** | - | - | - | - | - | - |
| *Lactobacillus reuteri** | - | - | - | - | - | - |
| *Lactobacillus rhamnosus** | 0.000 ± 0.002 | 0.001 ± 0.002 | 0.354 | - | - | - |
| *Micrococcaceae* | 0.000 ± 0.000 | 0.000 ± 0.000 | 0.945 | 0.000 ± 0.000 | 0.000 ± 0.000 | 0.827 |
| *Moraxellaceae* | 0.000 ± 0.000 | 0.000 ± 0.001 | 0.607 | 0.000 ± 0.000 | 0.000 ± 0.000 | 0.973 |
| *Morganella morganii* | - | - | - | - | - | - |
| *Polyangiaceae* | - | - | - | - | - | - |
| *Proteus* | - | - | - | - | - | - |
| *Proteus vulgaris* | - | - | - | - | - | - |
| *Pseudomonadaceae* | 0.001 ± 0.002 | 0.000 ± 0.000 | 0.367 | - | - | - |
| *Pseudomonas** | 0.001 ± 0.002 | 0.000 ± 0.000 | 0.367 | - | - | - |
| *Roseburia* | 0.019 ± 0.029 | 0.010 ± 0.015 | 0.367 | 0.016 ± 0.018 | 0.015 ± 0.037 | 0.973 |
| *Ruminococcaceae* | 0.129 ± 0.098 | 0.125 ± 0.078 | 0.945 | 0.108 ± 0.067 | 0.134 ± 0.078 | 0.827 |
| *Streptococcus* | 0.011 ± 0.017 | 0.020 ± 0.038 | 0.354 | 0.010 ± 0.016 | 0.011 ± 0.038 | 0.827 |
| *Streptococcus sp.** | 0.009 ± 0.014 | 0.019 ± 0.038 | 0.295 | 0.007 ± 0.012 | 0.005 ± 0.001 | 0.827 |
| *Salmonella* | - | - | - | - | - | - |
| *Salmonella enterica** | - | - | - | - | - | - |
| *Xanthomonadaceae* | - | - | - | - | - | - |
| All Taxa | 0.403 ± 0.145 | 0.397 ± 0.165 | 0.945 | 0.387 ± 0.194 | 0.341 ± 0.038 | 0.973 |
|  | | | | | | |
|  | **Mean ± Standard Deviation** | | | | | |
| **Taxa** | **Men**  **(n = 103)** | | | **Women**  **(n = 49)** | | |
|  | **Sarcopenia** | **No Sarcopenia** | **p-adj** | **Sarcopenia** | **No Sarcopenia** | **p-adj** |
| *Alistipes* | 0.093 ± 0.083 | 0.096 ± 0.070 | 0.812 | 0.089 ± 0.077 | 0.068 ± 0.057 | 0.949 |
| *Bacteroides fragilis* | 0.021 ± 0.037 | 0.015 ± 0.033 | 0.639 | 0.027 ± 0.051 | 0.010 ± 0.027 | 0.644 |
| *Bifidobacterium bifidum* | - | - | - | - | - | - |
| *Bifidobacterium longum subsp. longum* | - | - | - | - | - | - |
| *Bifidobacterium scardovii* | - | - | - | - | - | - |
| *Blautia* | 0.011 ± 0.013 | 0,009 ± 0.009 | 0.865 | 0.011 ± 0.013 | 0.012 ± 0.014 | 0.951 |
| *Clostridiaceae 1* | 0.003 ± 0.021 | 0,005 ± 0.016 | 0.332 | 0.016 ± 0.079 | 0.005 ± 0.017 | 0.949 |
| *Clostridioides difficile* | - | - | - | - | - | - |
| *Clostridium perfringens* | - | - | - | - | - | - |
| *Dermabacteraceae* | - | - | - | - | - | - |
| *Desulfovibrio desulfuricans* | - | - | - | - | - | - |
| *Eggerthella* | 0.000 ± 0.001 | 0.000 ± 0.000 | 0.812 | 0.000 ± 0.000 | 0.000 ± 0.000 | 0.951 |
| *Eggerthella YY918** | - | - | - | - | - | - |
| *Enterobacter** | - | - | - | - | - | - |
| *Enterobacteriaceae* | 0.071 ± 0.100 | 0.051 ± 0.063 | 0.812 | 0.057 ± 0.094 | 0.068 ± 0.076 | 0.644 |
| *Enterococcus faecium* | 0.002 ± 0.015 | 0.009 ± 0.050 | 0.639 | 0.024 ± 0.124 | 0.037 ± 0.169 | 0.674 |
| *Escherichia Shigella** | 0.052 ± 0.081 | 0.033 ± 0.051 | 0.639 | 0.043 ± 0.088 | 0.050 ± 0.063 | 0.644 |
| *Klebsiella* | 0.010 ± 0.049 | 0.016 ± 0.042 | 0.812 | 0.011 ± 0.039 | 0.002 ± 0.005 | 0.951 |
| *Klebsiella pneumoniae** | 0.010 ± 0.049 | 0.015 ± 0.040 | 0.812 | 0.010 ± 0.037 | 0.001 ± 0.005 | 0.951 |
| *Lachnoclostridium* | 0.007 ± 0.018 | 0.006 ± 0.010 | 0.812 | 0.005 ± 0.010 | 0.006 ± 0.010 | 0.951 |
| *Lactobacillus* | 0.018 ± 0.050 | 0.004 ± 0.010 | 0.812 | 0.032 ± 0.097 | 0.022 ± 0.058 | 0.951 |
| *Lactobacillus fermentum** | 0.001 ± 0.008 | 0.001 ± 0.002 | 0.982 | 0.000 ± 0.001 | 0.001 ± 0.003 | 0.951 |
| *Lactobacillus plantarum** | - | - | - | - | - | - |
| *Lactobacillus reuteri** | - | - | - | - | - | - |
| *Lactobacillus rhamnosus** | 0.000 ± 0.001 | 0.000 ± 0.000 | 0.812 | 0.000 ± 0.001 | 0.001 ± 0.003 | 0.644 |
| *Micrococcaceae* | 0.000 ± 0.000 | 0.000 ± 0.000 | 0.527 | 0.000 ± 0.000 | 0.000 ± 0.000 | 0.644 |
| *Moraxellaceae* | 0.000 ± 0.000 | 0.000 ± 0.001 | 0.978 | 0.000 ± 0.000 | 0.000 ± 0.001 | 0.692 |
| *Morganella morganii* | - | - | - | - | - | - |
| *Polyangiaceae* | - | - | - | - | - | - |
| *Proteus* | - | - | - | - | - | - |
| *Proteus vulgaris* | - | - | - | - | - | - |
| *Pseudomonadaceae* | 0.000 ± 0.002 | 0.000 ± 0.000 | 0.639 | 0.001 ± 0.002 | 0.000 ± 0.000 | 0.644 |
| *Pseudomonas** | 0.000 ± 0.002 | 0.000 ± 0.000 | 0.639 | 0.001 ± 0.002 | 0.000 ± 0.000 | 0.644 |
| *Roseburia* | 0.018 ± 0.028 | 0.014 ± 0.021 | 0.812 | 0.016 ± 0.020 | 0.009 ± 0.013 | 0.644 |
| *Ruminococcaceae* | 0.124 ± 0.095 | 0.135 ± 0.086 | 0.812 | 0.115 ± 0.071 | 0.120 ± 0.073 | 0.951 |
| *Streptococcus* | 0.008 ± 0.014 | 0.011 ± 0.021 | 0.812 | 0.016 ± 0.021 | 0.024 ± 0.046 | 0.951 |
| *Streptococcus sp.** | 0.007 ± 0.011 | 0.007 ± 0.010 | 0.812 | 0.012 ± 0.018 | 0.023 ± 0.046 | 0.644 |
| *Salmonella* | - | - | - | - | - | - |
| *Salmonella enterica** | - | - | - | - | - | - |
| *Xanthomonadaceae* | - | - | - | - | - | - |
| All Taxa | 0.388 ± 0.153 | 0.371 ± 0.131 | 0.978 | 0.421 ± 0.190 | 0.383 ± 0.183 | 0.951 |
|  | | | | | | |

Supplementary Table S8 Correlation of the extracted taxa to the urease subunit alpha. P-values were adjusted (p-adj) for each group.

| -) Taxa was present in less than 10% of patients and excluded from further analysis,  *) only genus/family was used for the calculation of all taxa |
| --- |

|  | **Correlation coefficient (spearman)** | | | | | | | | | | | | | | | | | | | | | | | | |
| --- | --- | --- | --- | --- | --- | --- | --- | --- | --- | --- | --- | --- | --- | --- | --- | --- | --- | --- | --- | --- | --- | --- | --- | --- | --- |
| **Taxa** | **All patients**  **(n = 152)** | | **Cirrhotic patients**  **(n = 96)** | | | | **Non-cirrhotic patients**  **(n = 56)** | | | | | | **Men**  **(n = 103)** | | | | | | **Women**  **(n = 49)** | | | | | | |
|  | **rs** | **p-adj** | **rs** | | **p-adj** | | | **rs** | **p-adj** | | | | **rs** | | | **p-adj** | | | | **rs** | **p-adj** | | | | |
| *Alistipes* | -0.03 | 0.79 | 0.04 | 0.829 | | | -0.14 | | | | 0.57 | | 0.03 | | 0.87 | | | | -0.17 | | | | | 0.59 | |
| *Bacteroides fragilis* | 0.08 | 0.50 | 0.12 | 0.341 | | | -0.03 | | | | 0.93 | | 0.18 | | 0.13 | | | | -0.14 | | | | | 0.59 | |
| *Bifidobacterium bifidum* | - | | - | | | | - | | | | | | - | | | | | | - | | | | | | |
| *Bifidobacterium longum subsp. longum* | - | | - | | | | - | | | | | | - | | | | | | - | | | | | | |
| *Bifidobacterium scardovii* | - | | - | | | | - | | | | | | - | | | | | | - | | | | | | |
| *Blautia* | -0.02 | 0.79 | 0.01 | 0.968 | | | -0.07 | | | | 0.74 | | 0.08 | | | | 0.56 | | -0.22 | | | | 0.42 | | |
| *Clostridiaceae 1* | 0.08 | 0.50 | 0.10 | 0.462 | | | 0.04 | | | | 0.89 | | -0.02 | | | | 0.89 | | 0.28 | | | | 0.19 | | |
| *Clostridioides difficile* | - | | - | | | | - | | | | | | - | | | | | | - | | | | | | |
| *Clostridium perfringens* | - | | - | | | | - | | | | | | - | | | | | | - | | | | | | |
| *Dermabacteraceae* | - | | - | | | | - | | | | | | - | | | | | | - | | | | | | |
| *Desulfovibrio desulfuricans* | - | | - | | | | - | | | | | | - | | | | | | - | | | | | | |
| *Eggerthella* | 0.06 | 0.63 | 0.04 | 0.83 | | | 0.02 | | | | 0.94 | | 0.03 | | | | 0.87 | | 0.08 | | | | 0.81 | | |
| *Eggerthella YY918* | - | | - | | | | - | | | | | | - | | | | | | - | | | | | | |
| *Enterobacter ** | 0.27 | 0.003 | - | | | | 0.37 | | | | 0.02 | | - | | | | | | - | | | | | | |
| *Enterobacteriaceae* | 0.52 | 1.61x 10⁻¹⁰ | 0.46 | 2.30 x10^-6^ | | | 0.62 | | | | 2.88 x 10^-6^ | | 0.44 | | | | 1.95 x 10^-5^ | | 0.66 | | | | 4.46 x 10^-6^ | | |
| *Enterococcus faecium* | 0.17 | 0.07 | 0.18 | 0.20 | | | 0.13 | | | | 0.57 | | 0.17 | | | | 0.16 | | 0.17 | | | | 0.59 | | |
| *Escherichia Shigella** | 0.45 | 6.80 x 10^-10^ | 0.38 | 0.001 | | | 0.64 | | | | 2.88 x 10^-6^ | | 0.41 | | | | 6.97 x 10^-5^ | | 0.52 | | | | 0.001 | | |
| *Klebsiella* | 0.42 | 6.59 x 10^-7^ | 0.46 | 2.30 x10^-6^ | | | 0.29 | | | | 0.09 | | 0.42 | | | | 4.60 x 10^-5^ | | 0.40 | | | | 0.02 | | |
| *Klebsiella pneumoniae** | 0.415 | 6.59 x 10^-7^ | 0.46 | 2.30 x10^-6^ | | | 0.28 | | | | 0.09 | | 0.42 | | | | 4.60 x 10^-5^ | | 0.40 | | | | 0.02 | | |
| *Lachnoclostridium* | 0.022 | 0.79 | 0.05 | 0.83 | | | -0.08 | | | | 0.09 | | 0.04 | | | | 0.87 | | -0.01 | | | | 0.96 | | |
| *Lactobacillus** | 0.190 | 0.05 | 0.17 | 0.22 | | | 0.10 | | | | 0.69 | | 0.28 | | | | 0.01 | | 0.02 | | | | 0.93 | | |
| *Lactobacillus fermentum** | 0.252 | 0.005 | 0.24 | 0.05 | | | - | | | | | | 0.35 | | | | 0.001 | | 0.03 | | | | 0.90 | | |
| *Lactobacillus plantarum** | - | | - | | | | - | | | | | | - | | | | | | - | | | | | | |
| *Lactobacillus rhamnosus** | 0.029 | 0.79 | -0.004 | | 0.97 | - | | | | | | 0.02 | | 0.89 | | | | 0.06 | | | | 0.81 | | | |
| *Lactobacillus reuteri** | - | | - | | | | - | | | | | | - | | | | | | - | | | | | | |
| *Micrococcaceae* | 0.176 | 0.07 | 0.13 | | 0.33 | 0.22 | | | | 0.22 | | 0.25 | | 0.03 | | | | 0.07 | | | | 0.81 | | | |
| *Moraxellaceae* | 0.167 | 0.07 | 0.15 | | 0.29 | 0.20 | | | | 0.30 | | 0.18 | | 0.13 | | | | 0.15 | | | | 0.59 | | | |
| *Morganella morganii* | - | | - | | | | - | | | | | | - | | | | | | - | | | | | | |
| *Polyangiaceae* | - | | - | | | | - | | | | | | - | | | | | | - | | | | | | |
| *Proteus* | - | | - | | | | - | | | | | | - | | | | | | - | | | | | | |
| *Proteus vulgaris* | - | | - | | | | - | | | | | | - | | | | | | - | | | | | | |
| *Pseudomonadaceae* | 0.08 | 0.50 | 0.14 | | 0.30 | - | | | | | | 0.13 | | 0.27 | | | | -0.06 | | | | | | | 0.81 |
| *Pseudomonas** | 0.08 | 0.50 | 0.14 | | 0.30 | - | | | | | | 0.13 | | 0.27 | | | | -0.06 | | | | | | | 0.81 |
| *Roseburia* | -0.05 | 0.67 | -0.03 | | 0.86 | -0.09 | | | | 0.72 | | -0.01 | | 0.89 | | | | -0.11 | | | | | | | 0.71 |
| *Ruminococcaceae* | -0.07 | 0.54 | -0.13 | | 0.33 | 0.004 | | | | 0.98 | | -0.14 | | 0.24 | | | | 0.13 | | | | | | | 0.63 |
| *Streptococcus* | 0.37 | 1.18 x 10^-6^ | 0.35 | | 0.002 | 0.39 | | | | 0.02 | | 0.45 | | 1.95 x 10^-5^ | | | | 0.20 | | | | | | | 0.46 |
| *Streptococcus sp.** | 0.35 | 4.14 x 10^-5^ | 0.33 | | 0.005 | 0.34 | | | | 0.04 | | 0.45 | | 1.95 x 10^-5^ | | | | 0.15 | | | | | | | 0.59 |
| *Salmonella* | - | | - | | | | - | | | | | | - | | | | | | - | | | | | | |
| *Salmonella enterica** | - | | - | | | | - | | | | | | - | | | | | | - | | | | | | |
| *Xanthomonadaceae* | - | | - | | | | - | | | | | | - | | | | | | - | | | | | | |
| All Taxa | 0.38 | 6.57 x10^-6^ | 0.32 | | 0.01 | 0.44 | | | | 0.01 | | 0.36 | | 0.001 | | | | 0.41 | | | | | | | 0.02 |

# Supplementary References

## R Packages

### Data Processing

1. Wickham H, François R, Henry L, Müller K, Vaughan D (2023). _dplyr: A Grammar of Data Manipulation_. R package version 1.1.4, <https://CRAN.R-project.org/package=dplyr>.
2. Wickham H, Averick M, Bryan J, Chang W, McGowan LD, François R, Grolemund G, Hayes A, Henry L, Hester J, Kuhn M, Pedersen TL, Miller E, Bache SM, Müller K, Ooms J, Robinson D, Seidel DP, Spinu V, Takahashi K, Vaughan D, Wilke C, Woo K, Yutani H (2019). “Welcome to the tidyverse.” _Journal of Open Source Software_, *4*(43), 1686. doi:10.21105/joss.01686 <https://doi.org/10.21105/joss.01686>.
3. Ooms J (2024). _writexl: Export Data Frames to Excel 'xlsx' Format_. R package version 1.5.0, <https://CRAN.R-project.org/package=writexl>.
4. Wickham H, Bryan J (2023). _readxl: Read Excel Files_. R package version 1.4.3, <https://CRAN.R-project.org/package=readxl>.
5. Signorell A (2024). _DescTools: Tools for Descriptive Statistics_. R package version 0.99.55, <https://CRAN.R-project.org/package=DescTools>.
6. Kuhn M, Jackson S, Cimentada J (2022). _corrr: Correlations in R_. R package version 0.4.4, <https://CRAN.R-project.org/package=corrr>.

### Microbiome Analysis

1. phyloseq: An R package for reproducible interactive analysis and graphics of microbiome census data. Paul J. McMurdie and Susan Holmes (2013) PLoS ONE 8(4):e61217.
2. Chi Liu, Yaoming Cui, Xiangzhen Li, Minjie Yao. microeco: an R package for data mining in microbial community ecology. FEMS Microbiology Ecology, 2021, Volume 97, Issue 2, fiaa255.
3. Liu, C., Li, X., Mansoldo, F.R.P., An, J., Kou, Y., Zhang, X., Wang, J., Zeng, J.,Vermelho, A.B., Yao, M., 2022. Microbial habitat specificity largely affects microbial co-occurrence patterns and functional profiles in wetland soils. Geoderma 418, 115866.
4. Charif, D. and Lobry, J.R. (2007)

### Visualization

1. H. Wickham. ggplot2: Elegant Graphics for Data Analysis. Springer-Verlag New York, 2016.
2. Kassambara A (2023). _ggpubr: 'ggplot2' Based Publication Ready Plots_. R package version 0.6.0, <https://CRAN.R-project.org/package=ggpubr>.
3. Ahlmann-Eltze, C., & Patil, I. (2021). ggsignif: R Package for Displaying Significance Brackets for 'ggplot2'. PsyArxiv. doi:10.31234/osf.io/7awm6
4. Neuwirth E (2022). _RColorBrewer: ColorBrewer Palettes_. R package version 1.1-3, <https://CRAN.R-project.org/package=RColorBrewer>.
5. Nordmann, E., McAleer, P., Toivo, W., Paterson, H. & DeBruine, L. (2021). Data visualisation using R, for researchers who don't use R. Preprint.

## References Systematic Literature Search

Arvola, T., Laiho, K., Torkkeli, S., Mykkänen, H., Salminen, S., Maunula, L., et al. (1999). Prophylactic Lactobacillus GG reduces antibiotic-associated diarrhea in children with respiratory infections: a randomized study. *Pediatrics* 104, e64. doi: 10.1542/peds.104.5.e64

Bajaj, J. S., Sikaroodi, M., Shamsaddini, A., Henseler, Z., Santiago-Rodriguez, T., Acharya, C., et al. (2021). Interaction of bacterial metagenome and virome in patients with cirrhosis and hepatic encephalopathy. *Gut* 70, 1162–1173. doi: 10.1136/gutjnl-2020-322470

Blow, F., Gioti, A., Goodhead, I. B., Kalyva, M., Kampouraki, A., Vontas, J., et al. (2020). Functional Genomics of a Symbiotic Community: Shared Traits in the Olive Fruit Fly Gut Microbiota. *Genome Biol Evol* 12, 3778–3791. doi: 10.1093/gbe/evz258

de Castro, A. L. M., Vollú, R. E., Peixoto, R. S., Grigorevski-Lima, A. L., Coelho, R. R. R., Bon, E. P. S., et al. (2011). Cellulolytic potential of a novel strain of Paenibacillus sp. isolated from the armored catfish Parotocinclus maculicauda gut. *Braz J Microbiol* 42, 1608–15. doi: 10.1590/S1517-838220110004000048

Hamilton, A. L., Kamm, M. A., Ng, S. C., and Morrison, M. (2018). Proteus spp. as Putative Gastrointestinal Pathogens. *Clin Microbiol Rev* 31. doi: 10.1128/CMR.00085-17

Hu, J., Zhong, X., Yan, J., Zhou, D., Qin, D., Xiao, X., et al. (2020). High-throughput sequencing analysis of intestinal flora changes in ESRD and CKD patients. *BMC Nephrol* 21, 12. doi: 10.1186/s12882-019-1668-4

Hu, Y., Sanders, J. G., Łukasik, P., D’Amelio, C. L., Millar, J. S., Vann, D. R., et al. (2018). Herbivorous turtle ants obtain essential nutrients from a conserved nitrogen-recycling gut microbiome. *Nat Commun* 9, 964. doi: 10.1038/s41467-018-03357-y

Huët, M. A. L., Wong, L. W., Goh, C. B. S., Ong, K. S., Dwiyanto, J., Reidpath, D., et al. (2020). First reported case of Gilbertella persicaria in human stool: outcome of a community study from Segamat, Johor, Malaysia. *Braz J Microbiol* 51, 2067–2075. doi: 10.1007/s42770-020-00323-z

Inoue, T., Nakayama, J., Moriya, K., Kawaratani, H., Momoda, R., Ito, K., et al. (2018). Gut Dysbiosis Associated With Hepatitis C Virus Infection. *Clin Infect Dis* 67, 869–877. doi: 10.1093/cid/ciy205

Karnachuk, O. V, Ikkert, O. P., Avakyan, M. R., Knyazev, Y. V, N Volochaev, M., Zyusman, V. S., et al. (2021). Desulfovibrio desulfuricans AY5 Isolated from a Patient with Autism Spectrum Disorder Binds Iron in Low-Soluble Greigite and Pyrite. *Microorganisms* 9. doi: 10.3390/microorganisms9122558

Kešnerová, L., Moritz, R., and Engel, P. (2016). Bartonella apis sp. nov., a honey bee gut symbiont of the class Alphaproteobacteria. *Int J Syst Evol Microbiol* 66, 414–421. doi: 10.1099/ijsem.0.000736

Kongrueng, J., Srinitiwarawong, K., Nishibuchi, M., Mittraparp-Arthorn, P., and Vuddhakul, V. (2018). Characterization and CRISPR-based genotyping of clinical trh-positive Vibrio parahaemolyticus. *Gut Pathog* 10, 48. doi: 10.1186/s13099-018-0275-4

Lailaja, V. P., Sumithra, T. G., Reshma, K. J., Anusree, V. N., Amala, P. V, Kishor, T. G., et al. (2022). Characterization of novel L-asparaginases having clinically safe profiles from bacteria inhabiting the hemolymph of the crab, Scylla serrata (Forskål, 1775). *Folia Microbiol (Praha)* 67, 491–505. doi: 10.1007/s12223-022-00952-x

Lau, W. L., Chang, Y., and Vaziri, N. D. (2021). The consequences of altered microbiota in immune-related chronic kidney disease. *Nephrol Dial Transplant* 36, 1791–1798. doi: 10.1093/ndt/gfaa087

Neumann, A. P., and Suen, G. (2018). The Phylogenomic Diversity of Herbivore-Associated Fibrobacter spp. Is Correlated to Lignocellulose-Degrading Potential. *mSphere* 3. doi: 10.1128/mSphere.00593-18

Neuvonen, M.-M., Tamarit, D., Näslund, K., Liebig, J., Feldhaar, H., Moran, N. A., et al. (2016). The genome of Rhizobiales bacteria in predatory ants reveals urease gene functions but no genes for nitrogen fixation. *Sci Rep* 6, 39197. doi: 10.1038/srep39197

Paixão, T. A., Roux, C. M., den Hartigh, A. B., Sankaran-Walters, S., Dandekar, S., Santos, R. L., et al. (2009). Establishment of systemic Brucella melitensis infection through the digestive tract requires urease, the type IV secretion system, and lipopolysaccharide O antigen. *Infect Immun* 77, 4197–208. doi: 10.1128/IAI.00417-09

Rai, R., Saraswat, V. A., and Dhiman, R. K. (2015). Gut microbiota: its role in hepatic encephalopathy. *J Clin Exp Hepatol* 5, S29-36. doi: 10.1016/j.jceh.2014.12.003

Ramatla, T. A., Mphuthi, N., Ramaili, T., Taioe, M. O., Thekisoe, O. M. M., and Syakalima, M. (2020). Molecular detection of virulence genes in Salmonella spp. isolated from chicken faeces in Mafikeng, South Africa. *J S Afr Vet Assoc* 91, e1–e7. doi: 10.4102/jsava.v91i0.1994

Ranganathan, N., Patel, B. G., Ranganathan, P., Marczely, J., Dheer, R., Pechenyak, B., et al. (2006). In vitro and in vivo assessment of intraintestinal bacteriotherapy in chronic kidney disease. *ASAIO J* 52, 70–9. doi: 10.1097/01.mat.0000191345.45735.00

Regan, M. D., Chiang, E., Liu, Y., Tonelli, M., Verdoorn, K. M., Gugel, S. R., et al. (2022). Nitrogen recycling via gut symbionts increases in ground squirrels over the hibernation season. *Science* 375, 460–463. doi: 10.1126/science.abh2950

Ren, X., Cao, S., Akami, M., Mansour, A., Yang, Y., Jiang, N., et al. (2022). Gut symbiotic bacteria are involved in nitrogen recycling in the tephritid fruit fly Bactrocera dorsalis. *BMC Biol* 20, 201. doi: 10.1186/s12915-022-01399-9

Ryvchin, R., Dubinsky, V., Rabinowitz, K., Wasserberg, N., Dotan, I., and Gophna, U. (2021). Alteration in Urease-producing Bacteria in the Gut Microbiomes of Patients with Inflammatory Bowel Diseases. *J Crohns Colitis* 15, 2066–2077. doi: 10.1093/ecco-jcc/jjab101

Sayavedra, L., Li, T., Bueno Batista, M., Seah, B. K. B., Booth, C., Zhai, Q., et al. (2021). Desulfovibrio diazotrophicus sp. nov., a sulfate-reducing bacterium from the human gut capable of nitrogen fixation. *Environ Microbiol* 23, 3164–3181. doi: 10.1111/1462-2920.15538

Schimmel, P., Kleinjans, L., Bongers, R. S., Knol, J., and Belzer, C. (2021). Breast milk urea as a nitrogen source for urease positive Bifidobacterium infantis. *FEMS Microbiol Ecol* 97. doi: 10.1093/femsec/fiab019

Scully, E. D., Hoover, K., Carlson, J., Tien, M., and Geib, S. M. (2012). Proteomic analysis of Fusarium solani isolated from the Asian longhorned beetle, Anoplophora glabripennis. *PLoS One* 7, e32990. doi: 10.1371/journal.pone.0032990

Shen, T.-C. D., Albenberg, L., Bittinger, K., Chehoud, C., Chen, Y.-Y., Judge, C. A., et al. (2015). Engineering the gut microbiota to treat hyperammonemia. *J Clin Invest* 125, 2841–50. doi: 10.1172/JCI79214

Srinivasan, V. B., and Rajamohan, G. (2019). Genome analysis of urease positive Serratia marcescens, co-producing SRT-2 and AAC(6’)-Ic with multidrug efflux pumps for antimicrobial resistance. *Genomics* 111, 653–660. doi: 10.1016/j.ygeno.2018.04.001

Suzuki, K., Benno, Y., Mitsuoka, T., Takebe, S., Kobashi, K., and Hase, J. (1979). Urease-producing species of intestinal anaerobes and their activities. *Appl Environ Microbiol* 37, 379–82. doi: 10.1128/aem.37.3.379-382.1979

Teh, J. J., Berendsen, E. M., Hoedt, E. C., Kang, S., Zhang, J., Zhang, F., et al. (2021). Novel strain-level resolution of Crohn’s disease mucosa-associated microbiota via an ex vivo combination of microbe culture and metagenomic sequencing. *ISME J* 15, 3326–3338. doi: 10.1038/s41396-021-00991-1

Uriot, O., Galia, W., Awussi, A. A., Perrin, C., Denis, S., Chalancon, S., et al. (2016). Use of the dynamic gastro-intestinal model TIM to explore the survival of the yogurt bacterium Streptococcus thermophilus and the metabolic activities induced in the simulated human gut. *Food Microbiol* 53, 18–29. doi: 10.1016/j.fm.2015.05.007

Wang, T., Tian, X.-L., Xu, X.-B., Li, H., Tian, Y., Ma, Y.-H., et al. (2022). Dietary supplementation of probiotics fermented Chinese herbal medicine Sanguisorba officinalis cultures enhanced immune response and disease resistance of crucian carp (Carassius auratus) against Aeromonas hydrophila. *Fish Shellfish Immunol* 131, 682–696. doi: 10.1016/j.fsi.2022.10.046

Wegmann, U., Louis, P., Goesmann, A., Henrissat, B., Duncan, S. H., and Flint, H. J. (2014). Complete genome of a new Firmicutes species belonging to the dominant human colonic microbiota ('Ruminococcus bicirculans’) reveals two chromosomes and a selective capacity to utilize plant glucans. *Environ Microbiol* 16, 2879–90. doi: 10.1111/1462-2920.12217

Wilson, C. M., Aggio, R. B. M., O’Toole, P. W., Villas-Boas, S., and Tannock, G. W. (2012). Transcriptional and metabolomic consequences of LuxS inactivation reveal a metabolic rather than quorum-sensing role for LuxS in Lactobacillus reuteri 100-23. *J Bacteriol* 194, 1743–6. doi: 10.1128/JB.06318-11

Wilson, C. M., Loach, D., Lawley, B., Bell, T., Sims, I. M., O’Toole, P. W., et al. (2014). Lactobacillus reuteri 100-23 modulates urea hydrolysis in the murine stomach. *Appl Environ Microbiol* 80, 6104–13. doi: 10.1128/AEM.01876-14

Xie, F., Xu, L., Wang, Y., and Mao, S. (2021). Metagenomic Sequencing Reveals that High-Grain Feeding Alters the Composition and Metabolism of Cecal Microbiota and Induces Cecal Mucosal Injury in Sheep. *mSystems* 6, e0091521. doi: 10.1128/mSystems.00915-21

Xu, C., Jia, Q., Zhang, L., Wang, Z., Zhu, S., Wang, X., et al. (2020). Multiomics Study of Gut Bacteria and Host Metabolism in Irritable Bowel Syndrome and Depression Patients. *Front Cell Infect Microbiol* 10, 580980. doi: 10.3389/fcimb.2020.580980

Yuan, Y., Wang, X., Xu, X., Liu, Y., Li, C., Yang, M., et al. (2020). Evaluation of a Dual-Acting Antibacterial Agent, TNP-2092, on Gut Microbiota and Potential Application in the Treatment of Gastrointestinal and Liver Disorders. *ACS Infect Dis* 6, 820–831. doi: 10.1021/acsinfecdis.9b00374

Yukawa-Muto, Y., Kamiya, T., Fujii, H., Mori, H., Toyoda, A., Sato, I., et al. (2022). Distinct responsiveness to rifaximin in patients with hepatic encephalopathy depends on functional gut microbial species. *Hepatol Commun* 6, 2090–2104. doi: 10.1002/hep4.1954

Zhang, Z., Li, M., Zhang, X., Zheng, N., Zhao, S., and Wang, J. (2020). A Novel Urease Inhibitor of Ruminal Microbiota Screened through Molecular Docking. *Int J Mol Sci* 21. doi: 10.3390/ijms21176006

Zhang, Z., Zhai, H., Geng, J., Yu, R., Ren, H., Fan, H., et al. (2013). Large-scale survey of gut microbiota associated with MHE Via 16S rRNA-based pyrosequencing. *Am J Gastroenterol* 108, 1601–11. doi: 10.1038/ajg.2013.221

Zhao, Z. X., Tang, X. H., Jiang, S. L., Pang, J. Q., Xu, Y. Bin, Yuan, D. D., et al. (2022). Astragaloside IV improves the pharmacokinetics of febuxostat in rats with hyperuricemic nephropathy by regulating urea metabolism in gut microbiota. *Front Pharmacol* 13, 1031509. doi: 10.3389/fphar.2022.1031509

Zhukova, M., Sapountzis, P., Schiøtt, M., and Boomsma, J. J. (2022). Phylogenomic analysis and metabolic role reconstruction of mutualistic Rhizobiales hindgut symbionts of Acromyrmex leaf-cutting ants. *FEMS Microbiol Ecol* 98. doi: 10.1093/femsec/fiac084
